# Multilocus Phylogeography of the Treefrog *Scinax eurydice* (Anura, Hylidae) Reveals a Plio-Pleistocene Diversification in the Atlantic Forest

**DOI:** 10.1371/journal.pone.0154626

**Published:** 2016-06-01

**Authors:** Lucas Menezes, Clarissa Canedo, Henrique Batalha-Filho, Adrian Antonio Garda, Marcelo Gehara, Marcelo Felgueiras Napoli

**Affiliations:** 1 Programa de Pós-Graduação em Diversidade Animal, Instituto de Biologia, Universidade Federal da Bahia, Campus Universitário de Ondina, Rua Barão de Jeremoabo, 40170–115, Salvador, Bahia, Brasil; 2 Universidade Federal do Rio de Janeiro, Museu Nacional, Departamento de Vertebrados, Quinta da Boa Vista, s/n, 20940–040, Rio de Janeiro, Rio de Janeiro, Brasil; 3 Universidade Federal do Rio Grande do Norte, Centro de Biociências, Departamento de Botânica, Ecologia e Zoologia, Laboratório de Anfíbios e Répteis-LAR, Campus Universitário, Lagoa Nova, 59078–900, Natal, Rio Grande do Norte, Brasil; 4 Programa de Pós-Graduação em Sistemática e Evolução, Centro de Biociências, Universidade Federal do Rio Grande do Norte, Campus Universitário Lagoa Nova, 59078–900, Natal, Rio Grande do Norte, Brasil; 5 Museu de Zoologia, Departamento de Zoologia, Instituto de Biologia, Universidade Federal da Bahia, Campus Universitário de Ondina, Rua Barão de Jeremoabo, 40170–115, Salvador, Bahia, Brasil; SOUTHWEST UNIVERSITY, CHINA

## Abstract

We aim to evaluate the genetic structure of an Atlantic Forest amphibian species, *Scinax eurydice*, testing the congruence among patterns identified and proposed by the literature for Pleistocene refugia, microrefugia, and geographic barriers to gene flow such as major rivers. Furthermore, we aim to evaluate predictions of such barriers and refugia on the genetic structure of the species, such as presence/absence of dispersal, timing since separation, and population expansions/contractions. We sequenced mitochondrial and nuclear genetic markers on 94 tissue samples from 41 localities. We inferred a gene tree and estimated genetic distances using mtDNA sequences. We then ran population clustering and assignment methods, AMOVA, and estimated migration rates among populations identified through mtDNA and nDNA analyses. We used a dated species tree, skyline plots, and summary statistics to evaluate concordance between population’s distributions and geographic barriers and Pleistocene refugia. *Scinax eurydice* showed high mtDNA divergences and four clearly distinct mtDNA lineages. Species tree and population assignment tests supported the existence of two major clades corresponding to northeastern and southeastern Atlantic Forest in Brazil, each one composed of two other clades. Lineage splitting events occurred from late Pliocene to Pleistocene. We identified demographic expansions in two clades, and inexistent to low levels of migrations among different populations. Genetic patterns and demographic data support the existence of two northern Refuge and corroborate microrefugia south of the Doce/Jequitinhonha Rivers biogeographic divide. The results agree with a scenario of recent demographic expansion of lowland taxa. *Scinax eurydice* comprises a species complex, harboring undescribed taxa consistent with Pleistocene refugia. Two rivers lie at the boundaries among populations and endorse their role as secondary barriers to gene flow.

## Introduction

The Atlantic Forest (hereafter AF) harbors one of the richest, most threatened and unique biotas in the world [1]. A high degree of human interference (less than 12% of the original distribution remaining), high levels of endemism [567 vertebrates (2.1%) and over 8000 plants (2.7%)], and continuous rates of species descriptions characterize it as a biodiversity hotspot [1,2]. Despite these impressive numbers, little was known about the region's biogeographic history, and only recently a clearer picture about the origin, distribution, and relationships of its biota has arisen [3–11].

Biodiversity is not evenly distributed in the AF, with centers of endemism supported by faunal inventories, phylogenetic analyses and phylogeographic studies [4,11]. Phylogeographic breaks have been reported for a number of organisms, including bees [6], amphibians [12–14], reptiles [7,15], birds [8,16,17], bats [9], and plants [10]. The causes of such breaks have been attributed to Pleistocene climatic cycles, rivers acting as barriers, and/or and geomorphological dynamics. Hence, both vicariance promoted by geographic barriers and refuge dynamics prompted by climatic fluctuations have been implicated in the AF's biota diversification [12,15,16]. Notwithstanding, some phylogeographic studies involving birds pointed absence of genetic structure through these recovered breaks, as well as demographic stability during the Last Glacial Maximum [18,19].

Geographic barriers associated to neotectonic activity have been evoked to explain evolution of biota from the AF [20]. Biogeographic history and evolutionary processes in the AF were deeply influenced by river systems and mountain ranges originated by tectonics dated before Pleistocene, which determined genetic patterns recognized in some taxa [7,14,21]. For amphibians, speciation events have been explained by the renewal of the topography in southeastern Brazil during the Tertiary [22], primarily by the elevation that culminated in the Serra do Mar and Serra da Mantiqueira and depressions that caused the formation of rivers like the Paraíba do Sul and the Pomba-Muriaé (see [23]). However, recent phylogeographic studies focusing on the AF biota have shown that divergence events have been ongoing from the late Tertiary through the Pleistocene, evidencing the effects of more recent phenomena in shaping the current distribution of genetic diversity (eg. [12,15,20]).

Pleistocene climatic fluctuations played a central role in the stability, biodiversity distribution, and phylogeographic endemism in the Brazilian Atlantic Forest [3–5]. Forest models predict historical stability in central and northern Atlantic Forest, corroborating paleopalynological records, areas of endemism, and mtDNA diversity for several taxa [3]. Phylogeographic data for amphibians supported historically stable forest areas in the northeastern, central and southeastern [4], while phylogeographic endemism for 25 vertebrates in the Brazilian AF endorse two bioclimatic domains identified by distribution models for present and past climates [5]. Phylogeographic work has now supported the subdivision between southern, central, and northern Atlantic Forest biotas [4], indicating that cryptic lineages are likely to be identified in taxa spanning these regions, especially the climatically distinct northern and southern portions [5].

The treefrog *Scinax eurydice* is widely distributed in the Atlantic Forest and is a species that occupies the forest edge, being able to use temporary and/or permanent pounds on the outer margin and adjacent open areas in the forest for breeding [24–28]. Populations exhibit phenotypic variation throughout the species' range with four distinct morphotypes recognized based on external morphology (body size and shape), color patterns, and bioacoustics (M. F. Napoli *et al*. “unpublished work”). The wide geographic distribution allied with acoustic and morphological variation of the advertisement call provides an ideal scenario to test the congruence of genetic lineages with biogeographic domains and centers of endemism proposed for the AF.

Herein, we evaluate population structure and historical demography of *S*. *eurydice* in the AF based on one mitochondrial and three nuclear genes, using 94 tissue samples covering most of the species distribution. Specifically, we addressed the following questions: (i) how many genetically differentiated populations exist along the species distribution? (ii) how long ago did they diverge? and (iii) is there concordance between geographic distribution of genetically differentiated populations and divergence times with possible geographic barriers and/or Pleistocene climatic cycles?

## Material and Methods

### Population sampling

**Fig 1:**
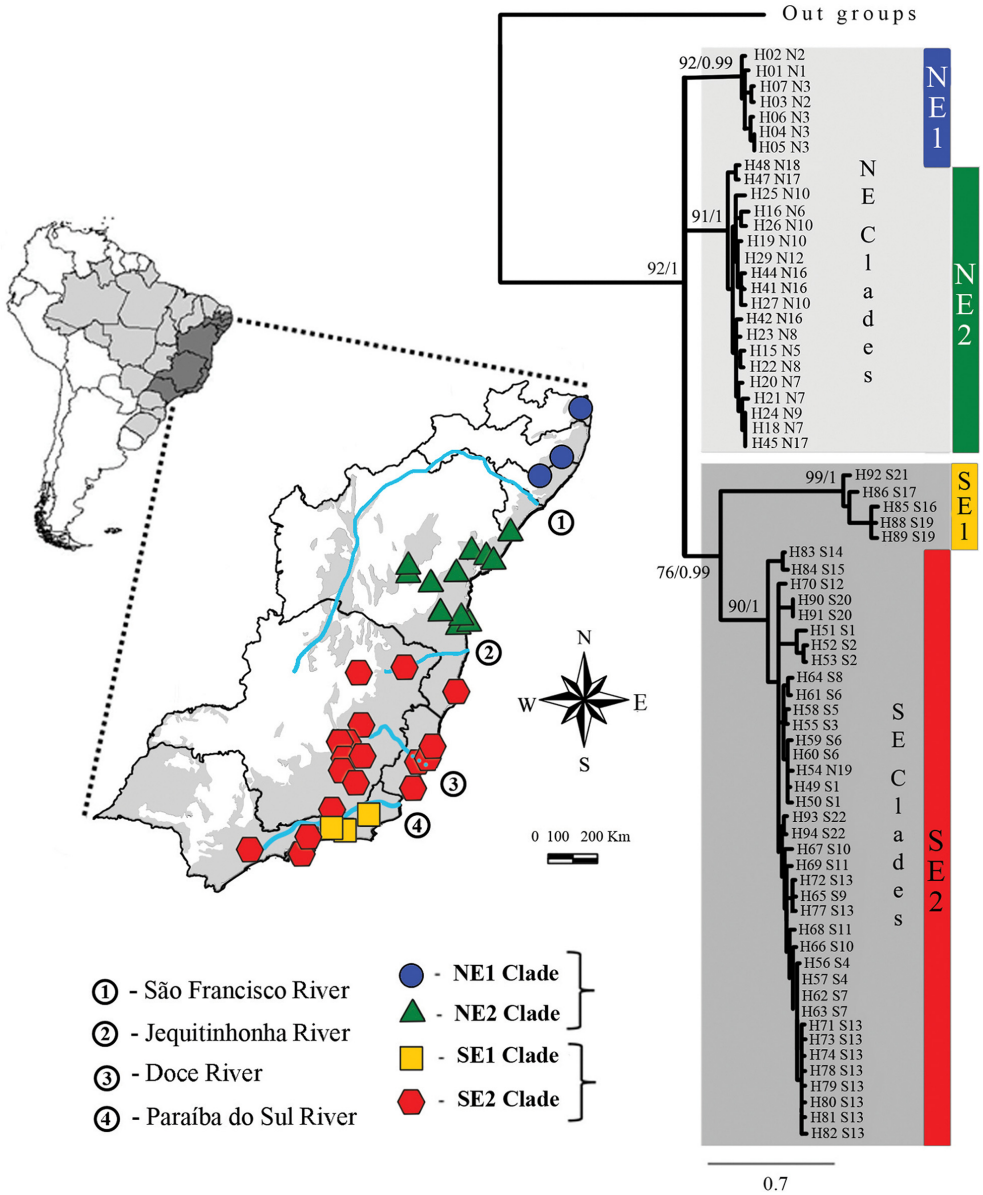
Localities sampled for mtDNA of *Scinax eurydice* and mitochondrial gene tree. The gene tree was obtained by Maximum Likelihood and Bayesian inference for the ND2 locus with support values (Bootstrap/Bayesian Posterior Probabilities) shown by major nodes. The blue circles, green triangles, red hexagons and yellow squares on the map indicate the locations sampled (mtDNA ND2) for each geographic group identified. The area shaded in dark gray on the Brazilian map represents states sampled for present work. The area in light gray in the inset represents the Atlantic Rainforest (based on SOS Mata Atlântica 2013). Haplotypes (H) represented on the tree topology are available in S1 Table. NE (Northeastern) and SE (Southeastern) correspond to populations identified. Numbers inside circles represent rivers listed as possible secondary barrier.

We obtained 94 tissue samples from 41 localities of *S*. *eurydice* from several Brazilian zoological collections, which cover most of the range of the species (Fig 1; S1 Table, see in Supporting Information). We used *S*. *x-signatus*, *S*. *auratus*, *S*. *nebulosus* and *S*. *berthae* as outgroups. Tissue samples for outgroups were obtained from Brazilian and Paraguayan zoological collections (see S1 Table).

### Molecular data collection

We amplified and sequenced fragments of one mitochondrial and three nuclear genes (see S1 Appendix): (1) NADH dehydrogenase subunit 2 (ND2, 969 bp); (2) partial coding sequence of the recombination activation gene (RAG1, 429 bp); (3) the β-fibrinogen intron 7 (β-fibint7, 321 bp); (4) and the 28S ribosomal RNA (28S, 796 bp). Primers used are listed in S2 Table in Supporting Infomtion. Electropherograms were assembled using CodonCode Aligner v. 3.5 (CodonCode Inc.). We aligned sequences using the CLUSTAL algorithm in MEGA5 [29] and used the PHI test in SPLITSTREE4 [30] to identify recombination in nuclear sequences. Loci with recombination were pruned and non-recombining regions were defined using the Fourgamete test in DnaSP 5.10.1 [31]. The gametic phases of nuclear genes were determined using PHASE algorithm [32] in DnaSP with default settings. Haplotypes with probabilities lower than 0.7 were excluded.

### Gene trees and mtDNA genetic distances

We estimated a gene tree for ND2 using Maximum Likelihood (ML) and Bayesian Inference (BI), as implemented inRAxMLGUI v.1.3 [33] and MrBayes 3 [34], respectively. Here and in all subsequent analyses substitution models were selected using JmodelTest2 [35] according to the AIC (Akaike Information Criterion). The ML analysis was performed with 100 runs and 1000 bootstrap replications (ML + 'thorough Bootstrap', 'n threads.' 4). For the BI analysis we performed 2 independent MCMC runs, each with four chains for 20 million steps sampling every 1000 generations and discarding the first two million as burn-in. Parameter convergence was checked in Tracer v1.6 (http://beast.bio.ed.ac.uk/Tracer). We calculated the genetic distance between recognized ND2 clades (net average distances—Da), using the Tamura-Nei distance [36] with gamma distribution in MEGA5. For each gene we also generated haplotype networks using the median-joining algorithm as implemented in Network 4.6.1.1 (available http://www.fluxus-engineering.com).

### Population assignment and basic population genetics statistics

We estimated the most likely number of groups (k) using the Bayesian clustering method implemented in Structure 2.3.3 [37]. The mitochondrial fragment was not used in this analysis. We assumed linkage model and independent allele frequencies. The analyses were repeated 10 times for each value of k from 1 to 10. Each k run consisted of six million MCMC generations with a burn-in of one million. To obtain the most likely number of k we used the Evannos’s K [38] method implemented in Structure Harvester [39] and the Bayes rule *Pr(K)* following [40]. Finally, ten runs with the most likely k were combined using Clumpp 1.1.2 [41] with the Greedy algorithm and 1000 permutations.

To determine the level of population structure among the defined groups we implemented an analysis of molecular variance (AMOVA) for mtDNA and nDNA separately, using the software ARLEQUIN 3.11 [42]. Localities with at least two individuals were assigned to populations. The significance was obtained using 1000 permutations. We calculated the following summary statistics for each defined group in DnaSP: haplotype diversity (Hd), nucleotide diversity (π), and their standard deviation (SD); Tajima‘s D and Fu’s F_s_ neutrality tests, and the population size change test Ramos-Onsins & Rozas R_2_ for each locus. The significances were calculated using 1000 coalescent simulations.

### Divergence time estimates

We estimated divergence times using the species tree method implemented in *BEAST 1.8.0 [43]. Furthermore, the Isolation with Migration method implemented in IMa2 [44] produces divergence time estimates, that were compared to *BEAST estimates.

We estimated the species tree by using *BEAST at the CIPRES Science Gateway [45]. This method estimates divergence time, population size change through time and species tree topology. Species assignment followed results of population assignment test (see section above and results for details). We used the strict clock model for all gene partition. We fixed ND2 substitution rate at 0.00957 mutation/site/million years [46]. For the nuclear loci substitution priors we used a default lognormal distribution (mean = 0 and sdtv = 1) and allowed the program to co-estimate rates. We implemented a piecewise linear and constant root and Yule Process as priors for the species tree model. We performed three independent MCMC runs of 200 million generations, sampling every 10,000 generations with a 10% burn-in. We checked for chain mixing, effective sample sizes (ESS), and convergence between runs using Tracer v1.6, making sure ESS values were higher than 200 for all parameters. The resulting trees were combined and summarized using TreeAnotator. We visualized the entire population of sampled trees in DensiTree 2.0.1 [47].

### Migration

IMa2 implements an Isolation-with-Migration model (IM) [44] and estimates effective population size, migration rate and divergence time. With few loci at hand it becomes hard to estimate all parameters of a four-population model [48]. Therefore, we ran a series of analyses of IM two-population model for all six pairwise combinations of the populations found [48]. We compare the two-population model results with a four-population model analysis with reduced number of parameters (migration only among current populations, ancestral populations had zero migration; exponential prior distribution with mean 0.6 for the migration parameter).

For each two-population analysis we ran IMa2 in M-mode with 20 chains and a geometric heating model (first parameter: 0.96; second parameter: 0.9), sampling 200,000 genealogies with a burn-in of 500,000 steps. We assumed a HKY substitution model for ND2, RAG1 and β-fibint7; and an Infinite Site model (IS) for 28S. Upper prior limits for population parameters were defined following the IMa2 manual (q = 16, t = 6.2, m = 0.6). For the four population model we ran IMa2 in M-mode with 40 chains and geometric heating model (first parameter: 0.975; second parameter: 0.75) and tree topology according to Species tree results. We sampled 100,000 genealogies after a burn-in of 1,000,000 steps. A HKY model was assumed for all genes. In all IMa2 analysis we accessed appropriate chain mixing by ensuring convergent results among 4 replicates with different seed numbers, and by checking the burntrend plot. Migration among populations was verified according to the likelihood ratio test (LRT), which accesses if the posterior distribution of the migration parameter significantly departs from zero. Estimated parameters were transformed to demographic quantities using the geometric mean of the mutation rates estimated by the Species tree analysis (see above). Posterior distribution graphs were generated using R programing environment [49] and ggplot2 R-package [50].

### Historical demography and population sizes

To evaluate the population size changes through time we used the Extended Bayesian Skyline Plot method (EBSP) in *BEAST v1.8.0 applied separately for each population identified by gene tree analysis. Two independent runs of EBSP were obtained with the following parameters: an initial tree generated by UPGMA, linear model, 200 million steps, parameters sampled every 10,000 steps and 10% burn-in (if necessary, the values of burn-in were changed to suit the ESS values > 200). We used an uncorrelated lognormal distribution for the mitochondrial fragment and a strict clock for nuclear fragments. To calibrate the EBSP we used the same strategy implemented in the *BEAST analysis (see section above). Parameter convergence between runs and performance analysis (considered ESS > 200) were conducted in Tracer v1.6 (http://beast.bio.ed.ac.uk/Tracer). We also obtained population size estimates from the IMa2 analysis (see migration section above).

## Results

### Gene trees and mtDNA genetic distance

We obtained 69 ND2 sequences (969 bp, 35 localities) comprising 49 haplotypes. ML and BI mitochondrial gene trees (GTR+G model) confirmed the monophyly of *S*. *eurydice* (Fig 1). Three clades were recovered, NE1, NE2 and SE, corresponding to northeastern and southeastern Brazil separated by the Jequitinhonha River (NE1+ NE2 and SE) and the São Francisco River (NE1 and NE2) (Fig 1). The NE1 clade includes samples from north of the São Francisco River and the NE2 clade comprises samples between the rivers São Francisco and Jequitinhonha. The SE present subclades: SE1 encompassing frogs from Rio de Janeiro State and SE2 including samples above the Jequitinhonha River. Genetic distances between mitochondrial clades ranged from 8% to 16% (Table 1). For nuclear genes we sequenced 91 samples for RAG1 (429 bp), 94 samples for 28S (768bp), and 58 samples for β-fibint7. Phased nuclear sequences resulted in 158 alleles for RAG1, 98 for β-fibint7, and 188 for 28S with P > 0.7. Twenty-four alleles for RAG1 and eighteen for β-fibint7 had low probability with PHASE and were removed from further analysis. The PHI test excluded recombination for β -fibint7 and 28S (P = 0.2539 and P = 1.0, respectively), but not for RAG1 (p = 0.0432). We applied the Four Gamete test to RAG1 to identify the largest non-recombinant block. We identified 46 pairs of sites with four gametic types and a minimum of five recombinant events. For RAG1 three non-recombinant blocks with 64 bp, 113 bp and 136 bp were identified, of which the longest was used in further analyses. The best-fitted substitution models of nuclear genes were as follow: HKY for RAG1 and GTR for β-fibint7 and 28S.

**Table 1:** Tamura-Nei distance (Da) within and between clades recognized by genes trees (ND2).

Distance Da (%)				
Clades	Within clades	Between clades		
		NE1	NE2	SE2
NE1	0.6			
NE2	0.9	8		
SE2	1.3	13	14	
SE1	2.0	16	15	10

**Fig 2:**
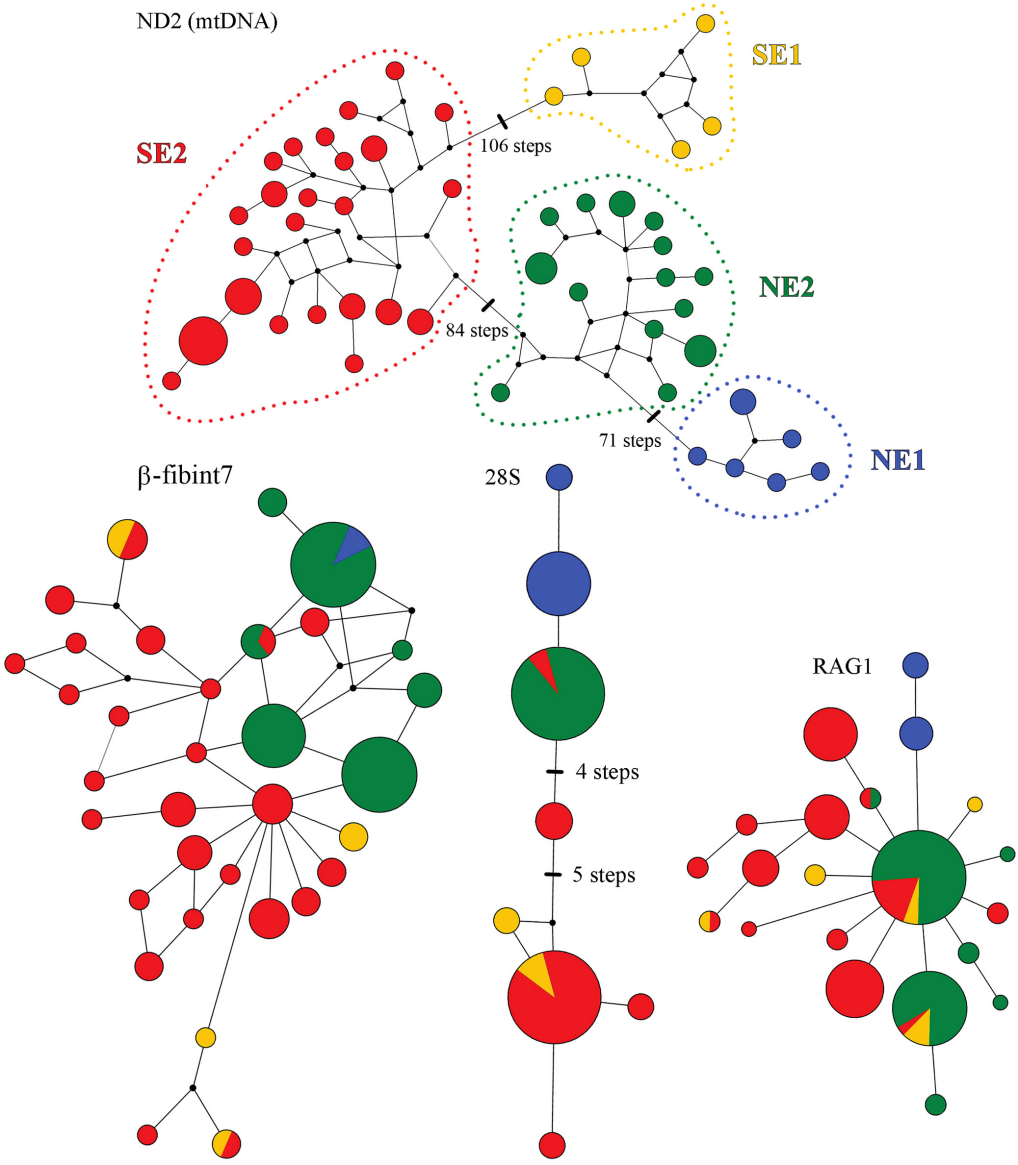
Median joining networks of the mitochondrial ND2 fragment and nuclear fragments RAG1, β-fibint7 and 28S. Black dots represent haplotypes not sampled. The colors of the observed haplotypes are based on the grouping of samples of mitochondrial clades (Fig 1): clade NE1 (blue circle), clade NE2 (green triangle), clade SE1 (yellow square), and clade SE2 (red hexagon). Branch lengths are proportional to the number of mutational steps.

Haplotype networks for nuclear loci showed variable levels of geographic structure (Fig 2). RAG1 showed lower geographic structure among populations than mitochondrial gene trees (ND2) and β-fibint7 showed a northeast-southeast distribution of haplotypes. In contrast, 28S haplotypes showed geographical structure concordant with major mitochondrial clades (NE, SE).

### Population assignment and basic population genetics statistics

**Fig 3:**
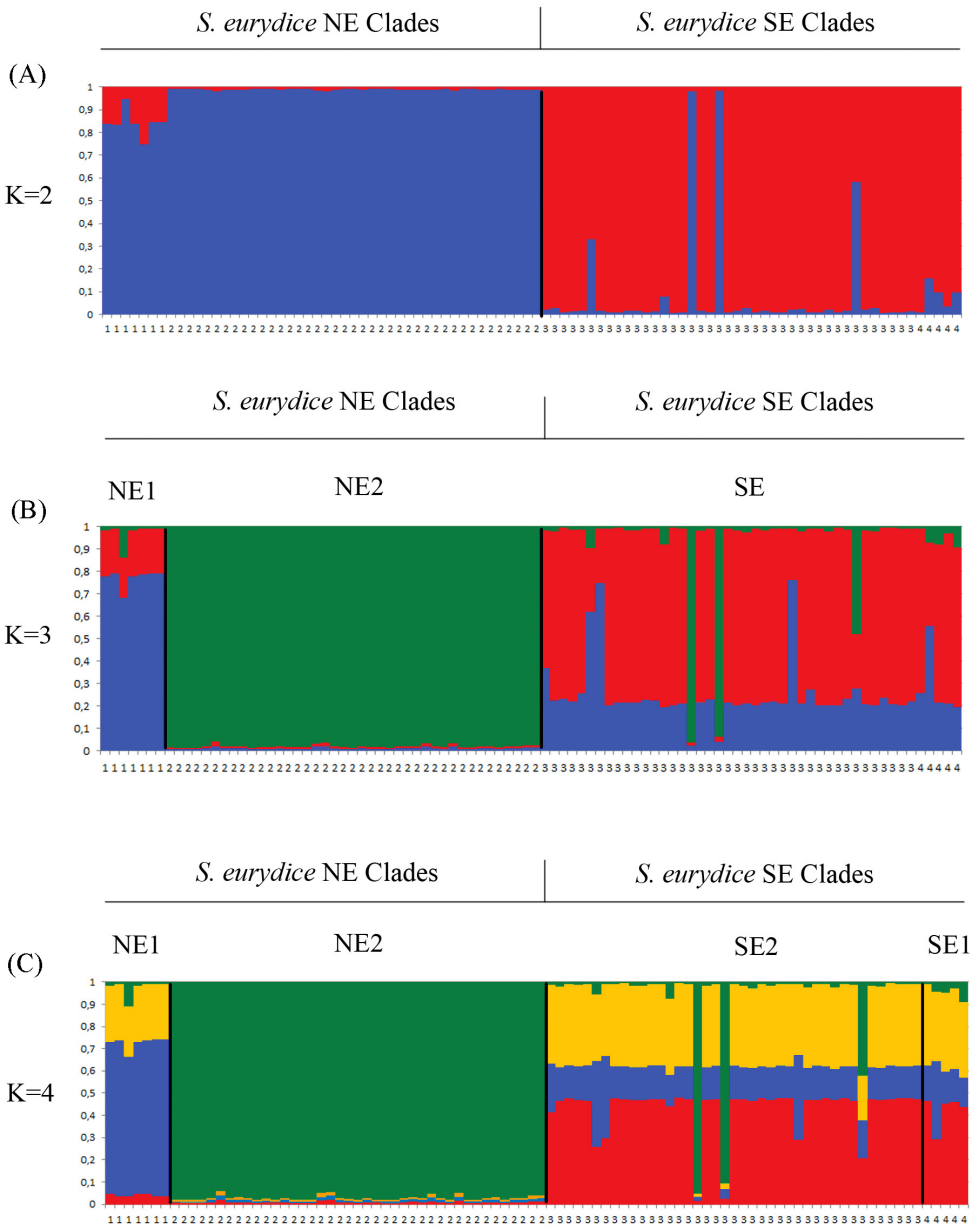
STRUCTURE plots of *Scinax eurydice* populations. Population structure between samples of northeast and southeast clades (A), northeast clades and southeast clade (B) and within of northeast and southeast mitochondrial clades (C) of *Scinax eurydice*. Plots shown represent the best K according to the ad hoc method after Princhard *et al*. (2000). Panels (A), (B) and (C) results from runs under the allele frequencies and admixture with correlated allele frequencies, respectively. Black lines delimit the mitochondrial clades.

For nuclear genes alone, the most likely number of populations according to Evanno's method was 2 (see S1 Fig in Supporting Information), with a second peak for k = 4. Structure tends to represent the most basal hierarchical structure in the dataset, and the second peak may represent a more refined structure of the dataset [38]. Indeed, Bayes rule [*Pr(k)*] recovered 4 as the best number of k (S3 Table). k = 2 shows a strong separation between northeastern (NE1 + NE2) and southeastern (SE1 + SE2) clades consistent with the basal separation recovered in the mitochondrial phylogeny (Fig 1). Q graphs for k = 4 do not match the mitochondrial phylogeny (Fig 1) and also exhibits admixture possibly due to gene flow (Fig 3, see Migration below).

Given the mitochondrial phylogeny, the k populations recovered by Evanno's method, and the number of populations selected through Bayes Rule, we chose to assign individuals to four populations. Because admixture in nuclear genes precludes us from unequivocally assigning individuals to four different populations, we used the mitochondrial phylogeny to assign individuals to four different populations.

The Amova results using these four populations indicate a significant genetic structure (groups defined as clades NE1, NE2, SE1, and SE2): (1) variation among groups = 89% and variation within groups = 6% for ND2; (2) variation among groups = 31% and variation within groups = 13% for RAG1; (3) variation among groups = 40% and variation within groups = 16% for β-fibint7 and (4) variation among groups = 82% and variation within groups = 17% for 28S (Table 2).

**Table 2:** Analyses of molecular variance (AMOVA) of *Scinax eurydice*.

Gene	Source of variation	DF	SQ	VC	PV
(A)	Among clades	3	1.503.859	4.665.168	89
	Among population within clades	14	167.779	323.912	6
	Within populations	39	98.117	251.581	5
	Total	56	1.769.754	5.240.661	
(B)	Among clades	3	27.825	0.28564	31
	Among population within clades	16	10.708	0.11653	13
	Within populations	116	59.525	0.51315	56
	Total	135	107.059	0.91532	
(C)	Among clades	3	35.253	0.71038	40
	Among population within clades	10	23.668	0.28477	16
	Within populations	64	50.182	0.78049	44
	Total	77	109.103	1.77924	
(D)	Among clades	3	294.444	2.89738	82
	Among population within clades	18	75.242	0.59786	17
	Within populations	142	6.015	0.04236	1
	Total	163	375.701	3.5760	

Mitochondrial DNA and nuclear DNA genes: (A) ND2, (B) RAG1, (C) β-fibint7 e (D) 28S. DF, degrees of freedom; SQ, sum of squares; VC, variance; PV, percentage of variation. All components of variance were significant at P < 0.05.

We found moderate (RAG1 56%, P < 0.00098; β-fibint7 44%, P < 0.00001) to low (28S 1%, P < 0.00001; ND2 5%, P < 0.00001) within-clade genetic divergences. Mitochondrial haplotype diversity ranged from 95% (± 9.6%) in NE1 to 99% (± 12.6) in SE1. Nucleotide diversity values for SE1 and SE2 were higher than NE1 and NE2 (Table 3). 28S showed small haplotype and nucleotide diversity with eight haplotypes for 94 specimens analyzed. RAG1 and β-fibint7 showed 21 and 31 haplotypes for 79 and 48 specimens analyzed, respectively.

**Table 3:** Diversity indexes for each fragment and clade.

Fragment	Locus	H	% Hd (SD)	% π (SD)	N	D	R_2_	Fs
ND2	All	49	98 (0.7)	7 (0.49)	69	-	-	-
	NE1	6	95 (9.6)	0.5 (0.09)	7	0.4840^ns^	0.1849^ns^	-1.002^ns^
	NE2	14	96 (3.1)	0.9 (0.09)	19	-1.1181^ns^	-0.0879*	-2.8525^ns^
	SE1	5	99 (12.6)	1.9 (0.41)	5	0.2307^ns^	0.1647^ns^	0.4484^ns^
	SE2	24	96 (2.1)	1.3 (0.15)	38	-1.3794*	0.072*	-3.3063^ns^
RAG1	All	21	81 (2.4)	0.57 (0.01)	158	-	-	-
	NE1	-	53 (1.5)	0.1 (0.01)	8	1.1665^ns^	0.2679^ns^	0.8664^ns^
	NE2	-	53 (4.9)	0.21 (0.03)	74	-1.3057*	0.0569^ns^	-2.9375*
	SE1	-	84 (8.1)	0.51 (0.14)	10	-0.9727^ns^	0.1674^ns^	-1.0835^ns^
	SE2	-	86 (1.9)	0.7 (0.05)	66	-0.5436^ns^	0.0902^ns^	-2.8701^ns^
β-fibint7	All	31	92 (0.01)	0.9 (0.05)	98	-	-	-
	NE1	-	np	np	2	Np	np	Np
	NE2	-	77 (3.5)	0.51 (0.02)	50	1.1255^ns^	0.1645^ns^	-1.1691^ns^
	SE1	-	67 (2.4)	1.2 (0.38)	6	2.1563^ns^	0.3333^ns^	-3.5264^ns^
	SE2	-	97 (1.2)	1.1 (0.1)	40	-0.4040^ns^	0.1029^ns^	-15.207*
28S	All	8	67 (2.3)	0.5 (0.01)	188	-	-	-
	NE1	-	26 (13.6)	0.1 (0.03)	14	-0.43764^ns^	0.1319*	1.251^ns^
	NE2	-	np	np	82	Np	np	np
	SE1	-	35 (15.9)	0.04 (0.02)	10	0.0150^ns^	0.1778^ns^	0.4167^ns^
	SE2	-	51 (6.1)	0.2 (0.05)	82	-0.3219^ns^	0.0882^ns^	1.3278^ns^

H, number of haplotypes; Hd, percentage of haplotype diversity and respective standard deviations (SD); π, percentage of nucleotide diversity and respective standard deviations; N, number of sequences; D, Tajima test; R2, Ramos-Onsins test; Fs, Fu test; np, no polymorphism; ns, no significant value;

*, P < 0.05.

Tamura-Nei distance (Da) within and between clades recognized by genes trees (ND2).

### Divergence times

**Fig 4:**
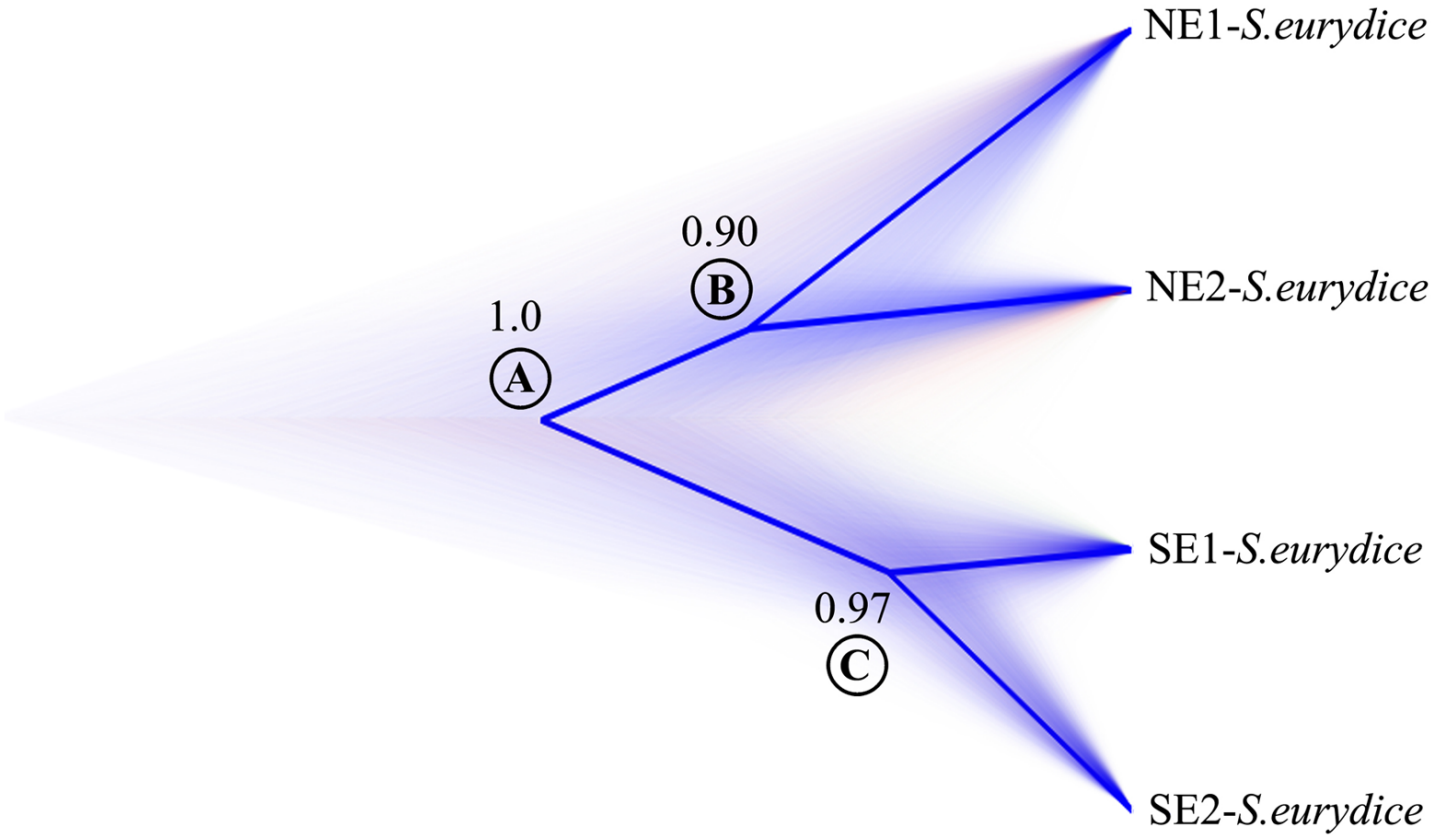
Chronogram of species tree from the combined mitochondrial and nuclear DNA data generated on *BEAST. Numbers by nodes represent clade posterior probabilities. Areas where many species trees agree on topology and/or branch lengths are densely colored in blue. The most frequent topologies are represented by shaded consensus tree. White circles with letters at nodes are divergence times and 95% (HPD) of high posterior density of divergence times between clades according to Table 4.

The *BEAST species tree based on the combined dataset (mtDNA and nDNA) was similar to the mitochondrial phylogeny. The basal split [NE and SE, Posterior Probability (PP) = 1.0] and the relationships among northern (NE1 and NE2, PP = 0.90) and southern populations (SE1 and SE2, PP = 0.97) were all strongly supported (Fig 4).

The dated chronogram generated in *BEAST revealed a recent history of diversification, with lineage splitting events occurring from late Pliocene to Pleistocene [3.9–0.4 Million of years (Myr); Fig 4; Table 4]. The oldest divergence event was the split between northeastern and southeastern clades around 2.1 Myr (95% high posterior density: 3.9–1.0 Myr). The youngest diversification event was the split of the southeastern clades (SE1 and SE2) around 0.8 Myr (95% high posterior density: 1.3–0.4 Myr). These results agree with divergence time estimates recovered in IMa2 (see S2 Appendix).

**Table 4:** Divergence times and confidence intervals [95% of high posterior density (HPD)].

Node	Time Ma (95% of HPD)
A	2.1 (1.0–3.9)
B	1.9 (0.7–2.1)
C	0.8 (0.4–1.3)

Analysis performed with combined dataset between lineages of *Scinax eurydice* estimated by BEAST. Node letters follow Fig 4.

### Migration

Mean values of migration rates for two-population and four-population models are largely in congruent. However, peak estimates for migration from SE2 to SE1 and from NE2 to SE1 are much lower in the four-population model (S4 Table, S3 and S4 Figs). One potential explanation is that for the two-population model, when there is migration with a third not included population, the IMa2 model is violated and migration rates can be biased [51]. Nevertheless, the exponential prior used for the migration parameter in the four-population model can also cause this difference in peak estimates. IMa two-population model analyses estimated significant bidirectional migration between populations NE2 and SE2; and populations SE1 and SE2, with higher population migration rates in the southward direction from NE2 to SE2, and from SE2 to SE1. Unidirectional migration was also significant from population NE2 to population SE1 (S4 Table, S3 Fig). No significant migration was found between population NE1 and any of the other populations identified here (S4 Table, S3 Fig). Likelihood ration tests for migration rates of the four-population model found significant migration between SE2 and NE2 with higher rates from NE2 to SE2; and unidirectional migration from SE2 to SE1 (S4 Table, S4 Fig).

### Historical demography and population sizes

Neutrality and population size change tests for ND2 indicated significant signals of demographic expansion in clades NE2 and SE2 (R2 for clade NE2, D and R2 for SE2). For RAG1 D and Fs tests were significant for samples comprising clade NE2 and for β-fibint7 only the Fs test was significant for samples that correspond to clade SE2. We did not find any evidence for demographic expansion in 28S (S3 Table). These results suggest that clades NE1 and SE1 did not experience events of demographic expansion.

**Fig 5:**
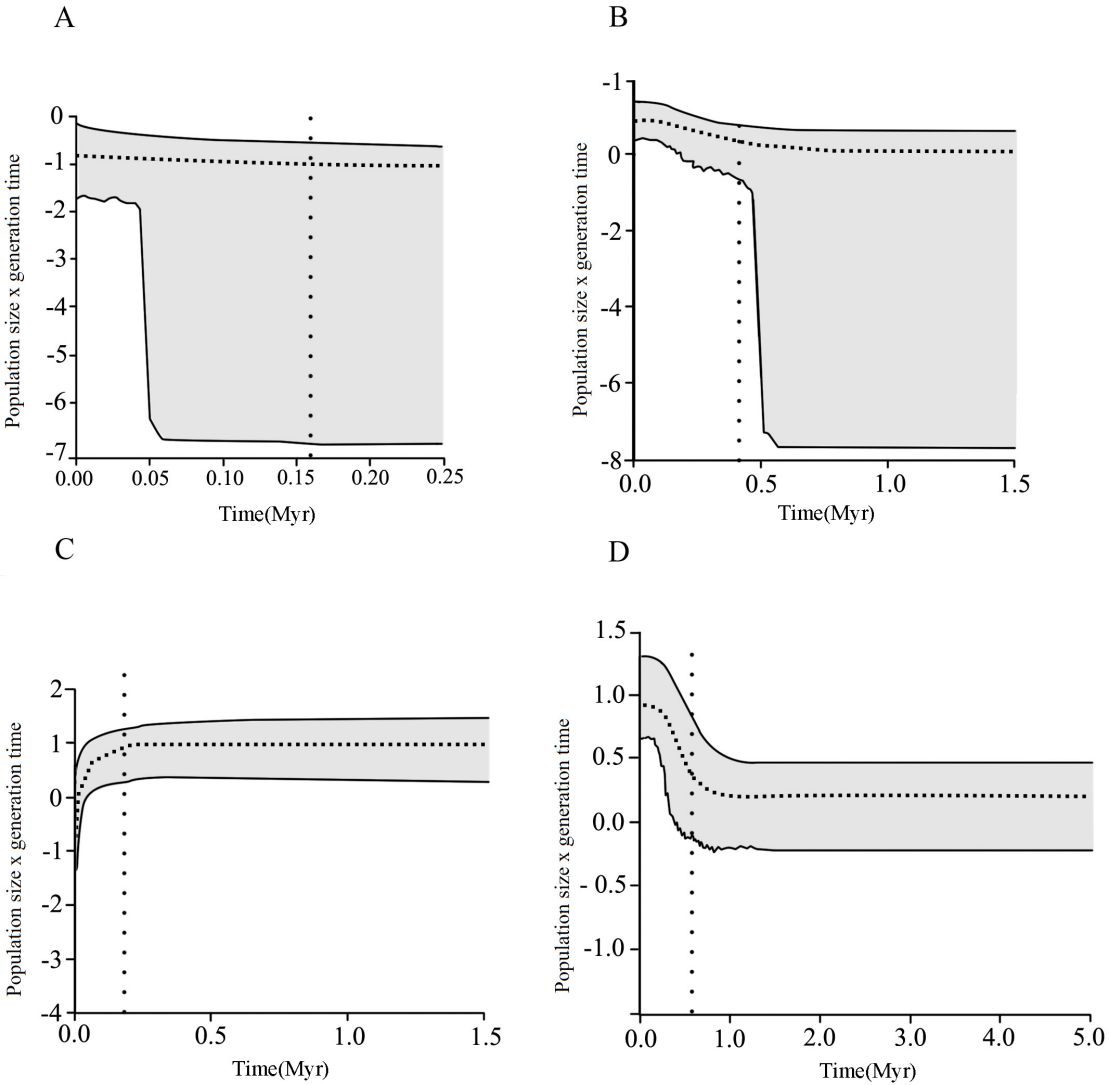
Extended Bayesian Skyline Plot (EBSP) results for populations of *Scinax eurydice*. The dotted horizontal line shows the median estimate of the EBSP and the gray area shows the upper and lower 95% highest posterior density (HPD). Each graph represents the EBSP analysis for: (A) clade NE1; (B) clade NE2; (C) SE1; (D) clade SE2. The dotted vertical line illustrates the approximate time when events of demographic expansion started in each clade of *S*. *eurydice*.

The EBSP evidenced that mtDNA clades of *S*. *eurydice* (NE1, NE2, SE1 and SE2) experienced distinct demographic histories. Changes in effective population sizes (Ne) through time were observed only for SE1 and NE2. We evidenced a reduction of Ne in clade SE1, which started about 130,000 years ago (Fig 5), in the late Pleistocene. On other hand, clade NE2 presented a demographic expansion starting about 400,000 years ago. In addition, we observed population growth in clade SE2, beginning 500,000 years ago.

IMa2 four-population model estimates of current population size suggest SE2 being the largest population with around 1.3 million individuals. NE1 represents the smallest population with around 170 thousand individuals. NE2 and SE1 have intermediate sizes with around 700 thousand individuals (S5 Fig). Two-population model estimates of current population sizes overlap four-population model estimates, but peak estimates suggest larger populations. This is expected if migration from a third population is not taken into account [48,51]. Ancestral population size estimates for two-population models suggest an ancestral population of 250 thousand individuals for NE1 and NE2 populations, and around 2 million individuals for SE1 and SE2 (S6 Fig). The same ancestral populations are poorly estimated in the four-population model, posterior distributions are rather flat with the ancestral of NE1 and NE2 having the highest probability at zero. The population size estimate of the ancestral of all for populations shows a peak at around 2.1 million individuals (S6 Fig).

## Discussion

Two different climatic domains based on congruent patterns of phylogeographic endemism for vertebrates have been recently proposed for the AF [5]. These domains are associated with broadly different climatic regimes, which divide the AF in two large northern and southern blocks, roughly at the Doce River. Our major break was found near the Jequitinhonha River, which reaches the Atlantic Ocean ~400 km north of the Doce River. Still, our sampling in the region between these two rivers is relatively poor, and because migrants from NE and SE populations across these rivers have been identified in other species studied [14], it is not possible to reject that the geographic separation between our clades lies actually near the Doce River. Nevertheless, other studies have also shown major breaks at the Jequitinhonha River valley for lizards [7], birds [8,16,52], and amphibians [4,14,53]. At last, the Doce and Jequitinhonha rivers are relatively close (considering the 3200 km North-South distribution of the AF, over 29° in latitude), and because they are not the barrier per se, representing in fact a climatic turnover, it is likely that the position of phylogeographic breaks will vary among clades because of the different, clade-specific responses to the presence of rivers.

Although not necessarily being the sole cause of such breaks, rivers can act as secondary barriers, partially restricting gene flow among populations [54]. This seems the case of the Doce River, which coincidently lies within a region of significant climatic turnover [5]. Still, examples of phylogeographic breaks for amphibians and reptiles along the AF that match the position of current rivers abound (e.g., [12,14,15,53,55]). This partial restriction is likely to be clade-specific and may be the source of differences among the exact position of phylogeographic breaks between SE and NE vertebrate clades identified in the literature. The geographic structure observed between *S*. *eurydice* mitochondrial clades may therefore be increased by the action of rivers as secondary barriers, restricting gene flow and promoting allopatry between the two northern clades (São Francisco River) and between northern and southern clades (Jequitinhonha River). Nevertheless, the signal for these barriers is not pervasive for all markers, and no river can be pointed as a barrier between the two southern mitochondrial clades.

Indeed, nuclear and mitochondrial markers frequently show different phylogeographic resolutions for the same biogeographic history due to differences in mutation rates, recombination, and effective population sizes [12,56]. Such differences are expected to occur more often in recently diverged populations or species. Accordingly, our nuclear markers supported the separation of NE and SE clades. Still, the two SE clades, although clearly distinct mitochondrially (average net distance of 10%, Table 1), were not fully recovered with nuclear genes. This may result from a secondary contact (with male biased migrations), incomplete lineage sorting, or both. The divergence observed for mitochondrial clades suggest that populations of a formally widespread species may have persisted in isolation for some time, likely in forest refugia and microrefugia (sensu [57]).

Microrefugia have been originally proposed to help explain the persistence in time of high-mountain species and communities in the *tepuis* of Venezuela, and their biogeographic significance for the establishment of present day biotas is undisputable [57]. In the AF, paleo modeling indicated two stable areas north of the Doce River, the Bahia and Pernambuco refuges, while areas south of the river have been predicted to be less stable [3]. Although such models match phylogeographic data for several taxa [5], they also contradict studies that have found high phylogeographic endemism and diversity on the southern portions of the AF [12,13,21,58,59]. Microrefugia may have contributed to the preservation of diversity at higher latitudes of the AF [5,13]. In fact, the same region where clade SE1 was found (Rio de Janeiro) has been shown to harbor endemic lineages consistent with the existence of a microrefugium during the Pleistocene [13,60].

Pleistocene climatic fluctuations have been implicated in the diversification of several animal taxa in the AF [4,8,16–19,61]. Cladogenetic events for *S*. *eurydice* exhibit spatial and temporal concordance with previous works, which recovered a range of temporal divergences between the Pliocene and Pleistocene in the AF [15,52]. Phylogeographic structure and divergence dates show that the separation between the mitochondrial clades of *S*. *eurydice* is consistent with allopatric diversification associated with Pliocene-Pleistocene climatic cycles. The two divergent clades in the Bahia and Pernambuco refuges that do not exchange migrants, the significant migration from NE to SE, and a highly divergent mitochondrial clade in high altitude areas at the southern portion of the AF support both refuges predicted north of the Doce River [3–5] and the existence of microrefugia at the southern portion of the AF [3,5,13]. Hence, a once widely distributed *S*. *eurydice* may have had its distribution fragmented and restricted to microrefuges in the southern AF and to large refuges in Pernambuco and Bahia, with a recent re-establishment of contact by a directional migration from climatically-stable northeastern populations to the southeast and from lowland southeastern populations (SE2) to upland populations (SE1).

During the Last Glacial Maximum rainforests were restricted to mountainous areas and high altitude species were less affected by cold and dry climates of Pleistocene glaciations [55]. Lowland species, in contrast, should have benefited from warmer climates and present signs of demographic expansions following the end of glaciations. Because southern populations of *S*. *eurydice* occupy less stable portions of the AF [3], they should present more prominent signals of demographic alterations. Indeed, SE1 and SE2 were the only populations showing significant contractions/expansions. However, SE1 presented a significant contraction while SE2 showed signals of demographic expansion. This contrasting result may reflect different adaptive niches for each population (and therefore different responses to the end of glaciations). Alternatively, the demographic expansion detected for SE2 may result from the high levels of migration from NE populations (which violate EBSP assumptions), the expansion of suitable lowland forested areas in warmer periods, or both. Dates recovered for these demographic fluctuations suggest a recent effect of temperature increasing for SE1 (~150–200k years ago), but a much older expansion for SE2 (~500–600k years ago). Nevertheless, demographic expansions within the same timeframe have been identified in this region of the AF for several other taxa, including frogs [14] and birds [18] suggesting the pervasive effects of historical events affecting several taxa in the region. Such scenario of different demographic responses along with high mitochondrial differentiation (lowest Da value of 8%), wide geographic distribution, and lack of migration among NE populations suggest that *S*. *eurydice* may actually represent more than one species. Future researches with *S*. *eurydice* populations using an integrative approach and inferences of hybridization in secondary contact zones could be used to describe and delimit the spatial patterns of variation in their geographic range in Atlantic Forest and provide a more accurate hypothesis for its taxonomy.

## Conclusions

*Scinax eurydice* is genetically structured across the Atlantic Forest, showing high mtDNA divergence and low to inexistent levels of migration among populations. Genetic patterns and demographic data match predictions for the existence of large Refugia in the Northern AF (Bahia and Pernambuco), as well as microrefugia south of the Doce/Jequitinhonha Rivers biogeographic division. Patterns of gene flow, demography, and divergence dates agree with a scenario of recent demographic expansion of lowland taxa, likely favored by the end of major glaciation events and the expansion of suitable habitats. Species tree, demographic parameters, temporal and spatial population structures recovered endorse *S*. *eurydice* as a complex of species. Two rivers lie at the boundaries among populations (Jequitinhonha and São Francisco), a pattern repeatedly found in the AF, which suggests that the role of rivers as secondary barriers must be re-evaluated. The current distribution of these populations and their origin are linked to the recent history of the AF, underscoring the importance of taxonomic reappraisals based on multiple lines of evidences to ensure the preservation not only of species, but of the evolutionary processes responsible for the origin of current biodiversity.

## Supporting Information

S1 Fig K values curve for K = 1–10 (Ln Pr (X/K)), and value of DK for K = 4. (PDF)

S2 Fig Posterior probability distributions of divergence parameter estimated by IMa2 transformed to million years. (A) Divergence estimates of the six two-population models. Each curve represents the estimated divergence time between two populations. (B) Divergence time estimates resulting from a four-population model. Each curve represents one of the three divergence parameters of a four-population model. Transformations were performed using the geometric mean of the mutation rates (7.52 x 10^−7^).
(PDF)

S3 Fig Posterior distribution of migration rate (effective number of gene copies) into each population estimated with a two-population model. (PDF)

S4 Fig Posterior distribution of migration rate (effective number of gene copies) into each population estimated with a four-population model. (PDF)

S5 Fig Current effective population size posterior distributions in number of individuals for the two-population and four-population models. Transformations were performed using the geometric mean of the mutation rates (7.52 x 10^−7^).
(PDF)

S6 Fig Ancestral effective population size posterior distributions in number of individuals for the two-population and four-population models. Transformations were performed using the geometric mean of the mutation rates (7.52 x 10^−7^).
(PDF)

S1 Table Tissue samples of *Scinax eurydice* used in the account: voucher number, locality, state, coordinates, sequences, haplotype code and locality code of specimens. Abbreviations of Brazilian states: PB, Paraíba; PE, Pernambuco; AL, Alagoas; SE, Sergipe; BA, Bahia; ES, Espírito Santo; MG, Minas Gerais; RJ, Rio de Janeiro; SP, São Paulo. Collection acronyms: CHUFPB, Coleção Herpetológica da Universidade Federal da Paraíba; URCA-H, Coleção Herpetológica da Universidade Regional do Cariri; UFBA, Museu de Zoologia da Universidade Federal da Bahia; CFBH; Coleção Célio F.B. Haddad, Departamento de Zoologia, Universidade Estadual Paulista “Júlio de Mesquita Filho”; MNRJ, Museu Nacional, Rio de Janeiro; JC-MTR, LSH-MTR and MTR, Coleção Miguel Trefaut Rodrigues, deposited at the Universidade de São Paulo; PUCMG, Coleção de Herpetologia do Museu de Ciências Naturais da Pontifícia Universidade Católica de Minas; UFMG; Coleção de Herpetologia da Universidade Federal de Minas Gerais; MZUFV, Museu de Zoologia João Moojen, Universidade Federal de Viçosa; IIBP-H; Colección Herpetológica del Instituto de Investigación Biológica del Paraguay, Asunción, Paraguay.
(PDF)

S2 Table Gene fragment, primer, sequence of primer and reference used in this study. (PDF)

S3 Table K run numbers, replicates, mean of log-likelihood and standard deviation of log-likelihood. (PDF)

S4 Table Parameters of posterior distributions of population migration rate estimation for two and four population models. * P < 0.05; ** P < 0.01; *** P < 0.001. (PDF)

S1 Appendix Laboratory procedures. (PDF)

S2 Appendix IMa2 Divergence Times Estimation Results. (PDF)

## References

[1] Myers N, Mittermeier RA, Mittermeier CG, da Fonseca GAB, Kent J. Biodiversity hotspots for conservation priorities. Nature. 2000;403:853–8. 10706275 10.1038/35002501

[2] Ribeiro MC, Metzger JP, Martensen AC, Ponzoni FJ, Hirota MM. The Brazilian Atlantic Forest: How much is left, and how is the remaining forest distributed? Implications for conservation. Biological Conservation. 2009;142:1141–1153.

[3] Carnaval AC, Moritz C. Historical climate modelling predicts patterns of current biodiversity in the Brazilian Atlantic forest. Journal of Biogeography. 2008;35:1187–1201.

[4] Carnaval AC, Hickerson MJ, Haddad CFB. Stability predicts genetic diversity in the Brazilian Atlantic Forest hotspot. Science. 2009;323:785–789. 10.1126/science.1166955 19197066

[5] Carnaval AC, Waltari E, Rodrigues MT, Rosauer D, VanDerWal J, Damasceno R, et al Prediction of phylogeographic endemism in an environmentally complex biome. Proceedings of the Royal Society B. 2014;281:20141461 10.1098/rspb.2014.1461 25122231 PMC4150330

[6] Batalha-Filho H, Waldschmidt AM, Campos LAO, Tavares MG, Fernandes-Salomão TM. Phylogeography and historical demography of the neotropical stingless bee *Melipona quadrifasciata* (Hymenoptera, Apidae): incongruence between morphology and mitochondrial DNA. Apidologie. 2010;41:534–547.

[7] Pellegrino KCM, Rodrigues MT, Waite A, Morando M, Yonenagayassuda Y, Sites JW Jr. Phylogeography and species limits in the *Gymnodactylus darwinii* complex (Gekkonidae, Squamata): Genetic structure coincides with river systems in the Brazilian Atlantic Forest. Biological Journal of Linnean Society. 2005;85:13–26.

[8] d'Horta FM, Cabanne GS, Meyer D, Miyaki CY. The genetic effects of Late Quaternary climatic changes over a tropical latitudinal gradient: Diversification of an Atlantic Forest passerine. Molecular Ecology. 2011;20:1923–1935. 10.1111/j.1365-294X.2011.05063.x 21410807

[9] Martins FM, Templeton AR, Pavan ACO, Kohlbach BC, Morgante JS. Phylogeography of the common vampire bat (*Desmodus rotundus*): marked population structure, Neotropical Pleistocene vicariance and incongruence between nuclear and mtDNA markers. BMC Evolutionary Biology. 2009;9:294 10.1186/1471-2148-9-294 20021693 PMC2801518

[10] Ribeiro RA, Lemos-Filho JP, Ramos ACS, Lovato MB. Phylogeography of the endangered rosewood *Dalbergia nigra* (Fabaceae): insights into the evolutionary history and conservation of the Brazilian Atlantic Forest. Heredity. 2011;106:46–57. 10.1038/hdy.2010.64 20517347 PMC3183853

[11] Vasconcelos TS, Prado VHM, da Silva FR, Haddad CFB. Biogeographic distribution patterns and their correlates in the diverse frog fauna of the Atlantic Forest hotspot. PLoS ONE. 2014;9:e104130 10.1371/journal.pone.0104130 25140882 PMC4139199

[12] Thomé MTC, Zamudio KR, Giovanelli JGR, Haddad CFB, Baldissera FA, Alexandrino J. Phylogeography of endemic toads and post-Pliocene persistence of the Brazilian Atlantic Forest. Molecular Phylogenetics and Evolution. 2010;55:1018–1031. 10.1016/j.ympev.2010.02.003 20139019

[13] Gehara M, Canedo C, Haddad CFB, Vences M. From widespread to microendemic: Molecular and acoustic analyses show that *Ischnocnema guentheri* (Amphibia: Brachycephalidae) is endemic to Rio de Janeiro, Brazil. Conservation Genetics. 2013;14:973–982.

[14] Tonini JFR, Costa LP, Carnaval AC. Phylogeographic structure is strong in the Atlantic Forest; predictive power of correlative paleodistribution models, not always. Journal of Zoological Systematics and Evolutionary Research. 2013;51:114–121.

[15] Grazziotin FG, Monzel M, Echeverrigaray S, Bonatto SL. Phylogeography of the *Bothrops jararaca* complex (Serpentes: Viperidae): Past fragmentation and island colonization in the Brazilian Atlantic Forest. Molecular Ecology. 2006;15:3969–3982. 17054497 10.1111/j.1365-294X.2006.03057.x

[16] Cabanne GS, d’Horta FM, Sari EHR, Santos FR, Miyaki CY. Nuclear and mitochondrial phylogeography of the Atlantic forest endemic *Xiphorhynchus fuscus* (Aves: Dendrocolaptidae): Biogeography and systematics implications. Molecular Phylogenetics and Evolution. 2008;49:760–773. 10.1016/j.ympev.2008.09.013 18849002

[17] Maldonado-Coelho M. Climatic oscillations shape the phylogeographical structure of Atlantic Forest fire-eye antbirds (Aves: Thamnophilidae). Biological Journal of the Linnean Society. 2012;105:900–924.

[18] Batalha-Filho H, Cabanne GS, Miyaki CY. Phylogeography of an Atlantic forest passerine reveals demographic stability through the last glacial maximum. Molecular Phylogenetics and Evolution. 2012;65:892–902. 10.1016/j.ympev.2012.08.010 22940152

[19] Cabanne GS, Sari EHR, Meyer D, Santos FR, Miyaki CY. Matrilineal evidence for demographic expansion, low diversity and lack of phylogeographic structure in the Atlantic forest endemic Greenish *Schiffornis schiffornis virescens* (Aves: Tityridae). Journal of Ornithology. 2013;154:371–384.

[20] Thomé MTC, Zamudio KR, Haddad CFB, Alexandrino J. Barriers, rather than refugia, underlie the origin of diversity in toads endemic to the Brazilian Atlantic Forest. Molecular Ecology. 2014;23:6152–6164. 10.1111/mec.12986 25363843

[21] Alvarez-Presas M, Sánchez-Gracia A, Carbayo F, Rozas J, Riutort M. Insights into the origin and distribution of biodiversity in the Brazilian Atlantic forest hotspot: a statistical phylogeographic study using a low-dispersal organism. Heredity. 2014;112:656–65. 10.1038/hdy.2014.3 24549112 PMC4023448

[22] Duellman WE. Distribution Patterns of Amphibians in South America In: Duellman WE, editor. Patterns of Distribution of Amphibians. Baltimore and London: The Johns Hopkins University; 1999 p. 255–327.

[23] Radambrasil Projeto. Folhas SF. 23/24 Rio de Janeiro/Vitória: geologia, geomorfologia, pedologia, vegetação e uso potencial da terra. Rio de Janeiro: Ministério das Minas e Energia, Secretaria Geral; 1983 p. 775.

[24] Pombal-Jr JP, Bastos RP, Haddad CFB. Vocalizações de algumas espécies do gênero *Scinax* (Anura, Hylidae) do Sudeste do Brasil e comentários taxonômicos. Naturalia. 1995;20:213–225.

[25] Hartmann MT. Geographic Distribution. *Scinax eurydice* (Maracás Snouted Treefrog). Herpetological Review. 2002;33:222–223.

[26] Feio R, Caramaschi U. Contribuição ao conhecimento da herpetofauna do nordeste do estado de Minas Gerais, Brasil. Phyllomedusa. 2002;1(2);105–111.

[27] Bastazini CV, Munduruca JFV, Rocha PLB, Napoli MF. Which environmental variables better explain changes in anuran community composition? A case study in the restinga of Mata de São João, Bahia, Brazil. Herpetologica. 2007;63(4):459–471.

[28] Feio RN, Santos PS, Cassini CS, Dayrell JS, Oliveira EF. Anfíbios da Serra do Brigadeiro-MG. MG.Biota. 2008;1(1):04–31.

[29] Tamura K, Peterson D, Peterson N, Stecher G, Nei M, Kumar S. MEGA5: Molecular evolutionary genetics analysis using maximum likelihood, evolutioanry distance, and maximum parsimony methods. Molecular Biology and Evolution. 2011;28:2731–2739. 10.1093/molbev/msr121 21546353 PMC3203626

[30] Huson DH, Bryant D. Application of phylogenetic networks in evolutionary studies. Molecular Biology and Evolution. 2006;23:254–267. 16221896 10.1093/molbev/msj030

[31] Librado P, Rozas J. DnaSP v5: A software for comprehensive analysis of DNA polymorphism data. Bioinformatics. 2009;25:1451–1452. 10.1093/bioinformatics/btp187 19346325

[32] Stephens M, Smith NJ, Donnelly P. A new statistical method for haplotype reconstruction from population data. American Journal of Human Genetics. 2001;68:978–989. 11254454 10.1086/319501 PMC1275651

[33] Stamatakis A. RAxML-VI-HPC: Maximum likelihood-based phylogenetic analyses with thousands of taxa and mixed models. Bioinformatics. 2006;22:2688–2690. 16928733 10.1093/bioinformatics/btl446

[34] Ronquist F, Huelsenbeck JP. MrBayes 3: Bayesian phylogenetic inference under mixed models. Bioinformatics. 2003;19:1572–1574. 12912839 10.1093/bioinformatics/btg180

[35] Darriba D, Taboada GL, Doallo R, Posada D. jModelTest 2: more models, new heuristics and parallel computing. Nature Methods. 2012;9: 772–772. 10.1038/nmeth.2109 PMC4594756 22847109

[36] Tamura K, Nei M. Estimation of the number of nucleotide substitutions in the control region of mitochondrial DNA in humans and chimpanzees. Molecular Biology and Evolution. 1993;10:512–526. 8336541 10.1093/oxfordjournals.molbev.a040023

[37] Pritchard JK, Stephens M, Donnelly P. Inference of population structure using multilocus genotype data. Genetics. 2000;155:945–959. 10835412 10.1093/genetics/155.2.945 PMC1461096

[38] Evanno G, Regnaut S, Goudet J. Detecting the number of clusters of individuals using the software STRUCTURE: A simulation study. Molecular Ecology. 2005;14:2611–2620. 15969739 10.1111/j.1365-294X.2005.02553.x

[39] Earl DA, vonHoldt BM. STRUCTURE HARVESTER: A website and program for visualizing STRUCTURE output and implementing the Evanno method. Conservation Genetics Resources. 2012 4:359–361.

[40] Pritchard JK, Wen X, Falush D. Documentation for structure software: Version 2.3. Department of Statistics, University of Oxford. [cited 2010 Feb 2]. Available from: http://pritchardlab.stanford.edu/structure_software/release_versions/v2.3.4/structure_doc.pdf. Accessed 25 July 2014.

[41] Jakobsson M, Rosenberg NA. CLUMPP: A cluster matching and permutation program for dealing with label switching and multimodality in analysis of population structure. Bioinformatics. 2007;23:1801–1806. 17485429 10.1093/bioinformatics/btm233

[42] Excoffier L, Smouse PE, Quattro JM. Analysis of molecular variance inferred from metric distances among DNA haplotypes: Application to human mitochondrial DNA restriction data. Genetics. 1992;131:479–491. 1644282 10.1093/genetics/131.2.479 PMC1205020

[43] Drummond AJ, Xie W, Heled J. Bayesian Inference of Species Trees from Multilocus Data Using *BEAST. Molecular Biology and Evolution. 2012;29:1969–1973. 19906793 10.1093/molbev/msp274 PMC2822290

[44] Hey J, Nielsen R. Multilocus methods for estimating population sizes, migration rates and divergence time, with applications to the divergence of *Drosophila pseudoobscura* and *D*. *persimilis*. Genetics. 2004;167:747–760. 15238526 10.1534/genetics.103.024182 PMC1470901

[45] Miller MA, Pfeiffer W, Schwartz T. Creating the CIPRES Science Gateway for inference of large phylogenetic trees. Gateway Computing Environments Workshop (GCE). 2010;1–8.

[46] Crawford AJ. Huge populations and old species of Costa Rican and Panamanian dirt frogs inferred from mitochondrial and nuclear gene sequences. Molecular Ecology. 2003;12:2525–2540. 12969459 10.1046/j.1365-294x.2003.01910.x

[47] Bouckaert RR. DensiTree: Making sense of sets of phylogenetic trees. Bioinformatics. 2010;26:1372–1373. 10.1093/bioinformatics/btq110 20228129

[48] Hey J. The divergence of chimpanzee species and subspecies as revealed in multipopulation isolation-with-migration analyses. Molecular Biology and Evolution. 2010;27:921–933. 10.1093/molbev/msp298 19955478 PMC2877540

[49] R Development Core Team. R: a language and environment for statistical computing. R Foundation for Statical Computing 2013 Avaliable: http://www.r-project.org. Accessed 25 July 2014.

[50] Wickham H. ggplot2—Elegant graphics for data analysis. 1st ed Springer; 2009; p. 2013.

[51] Strasburg JL, Rieseberg LH. How robust are “isolation with migration” analyses to violations of the im model? A simulation study. Molecular Biology and Evolution. 2010;27:297–310. 10.1093/molbev/msp233 19793831 PMC2877552

[52] Batalha-Filho H, Irestedt M, Fjeldså J, Ericson PGP, Silveira LF, Miyaki CY. Molecular systematics and evolution of the *Synallaxis ruficapilla* complex (Aves: Furnariidae) in the Atlantic Forest. Molecular Phylogenetics and Evolution. 2013;67:86–94. 10.1016/j.ympev.2013.01.007 23340003

[53] Brunes TO, Alexandrino J, Baêta D, Zina J, Haddad CFB, Sequeira F. Species limits, phylogeographic and hybridization patterns in Neotropical leaf frogs (Phyllomedusinae). Zoologica Scripta. 2014;43:586–604.

[54] Fouquet A, Ledoux JB, Dubut V, Noonan BP, Scotti I. The interplay of dispersal limitation, rivers, and historical events shapes the genetic structure of an Amazonian frog. Biological Journal of the Linnean Society. 2012;106:356–373.

[55] Amaro RC, Rodrigues MT, Yonenaga-Yassuda Y, Carnaval AC. Demographic processes in the montane Atlantic rainforest: Molecular and cytogenetic evidence from the endemic frog *Proceratophrys boiei*. Molecular Phylogenetics and Evolution. 2012;62:880–888. 10.1016/j.ympev.2011.11.004 22108674

[56] Toews DPL, Brelsford A. The biogeography of mitochondrial and nuclear discordance in animals. Molecular Ecology. 2012;21:3907–3930. 10.1111/j.1365-294X.2012.05664.x 22738314

[57] Rull V. Microrefugia. Journal of Biogeography. 2009;36:481–484.

[58] Clemente-Carvalho RBG, Klaczko J, Perez SI, Alves ACR, Haddad CFB, Sérgio F. Molecular phylogenetic relationships and phenotypic diversity in miniaturized toadlets, genus *Brachycephalus* (Amphibia: Anura: Brachycephalidae). Molecular Phylogenetics and Evolution. 2011;61:79–89. 10.1016/j.ympev.2011.05.017 21693192

[59] Fusinatto LA, Alexandrino J, Haddad CFB, Brunes TO, Rocha CFD, Sequeira F. Cryptic genetic diversity is paramount in small-bodied amphibians of the genus *Euparkerella* (Anura: Craugastoridae) endemic to the Brazilian Atlantic forest. PLoS ONE. 2013;8:1–12. 10.1371/journal.pone.0079504 PMC3815154 24223956

[60] Gehara M, Crawford AJ, Orrico VGD, Rodríguez A, Lötters S, Fouquet A, et al High levels of diversity uncovered in a widespread nominal taxon: continental phylogeography of the Neotropical tree frog *Dendropsophus minutus*. PLoS ONE. 2014;9:e103958 10.1371/journal.pone.0103958 25208078 PMC4160190

[61] Carnaval AC, Bates JM. Amphibian DNA shows marked genetic structure and tracks Pleistocene climate change in northeastern Brazil. Evolution. 2007;61: 2942–2957. 17941838 10.1111/j.1558-5646.2007.00241.x

