# Supplementary figures and images for: Multilocus Phylogeography of the Treefrog Scinax eurydice (Anura, Hylidae) Reveals a Plio-Pleistocene Diversification in the Atlantic Forest
Source: PLoS One. 2016 Jun 1;11(6):e0154626. doi: 10.1371/journal.pone.0154626 (PMC4889069; doi:10.1371/journal.pone.0154626)

$$\text{DeltaK} = \text{mean}(|L''(K)|) / \text{sd}(L(K))$$

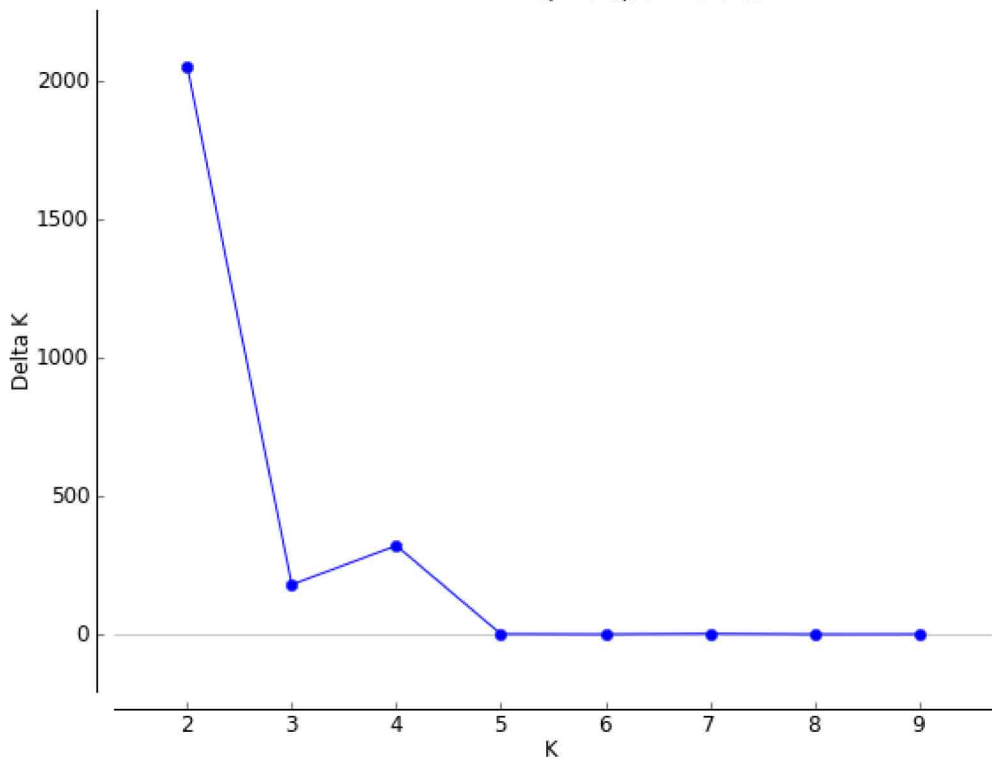

Supplement: S1 Fig — (PDF) [file pone.0154626.s001.pdf]

t posterior distribution

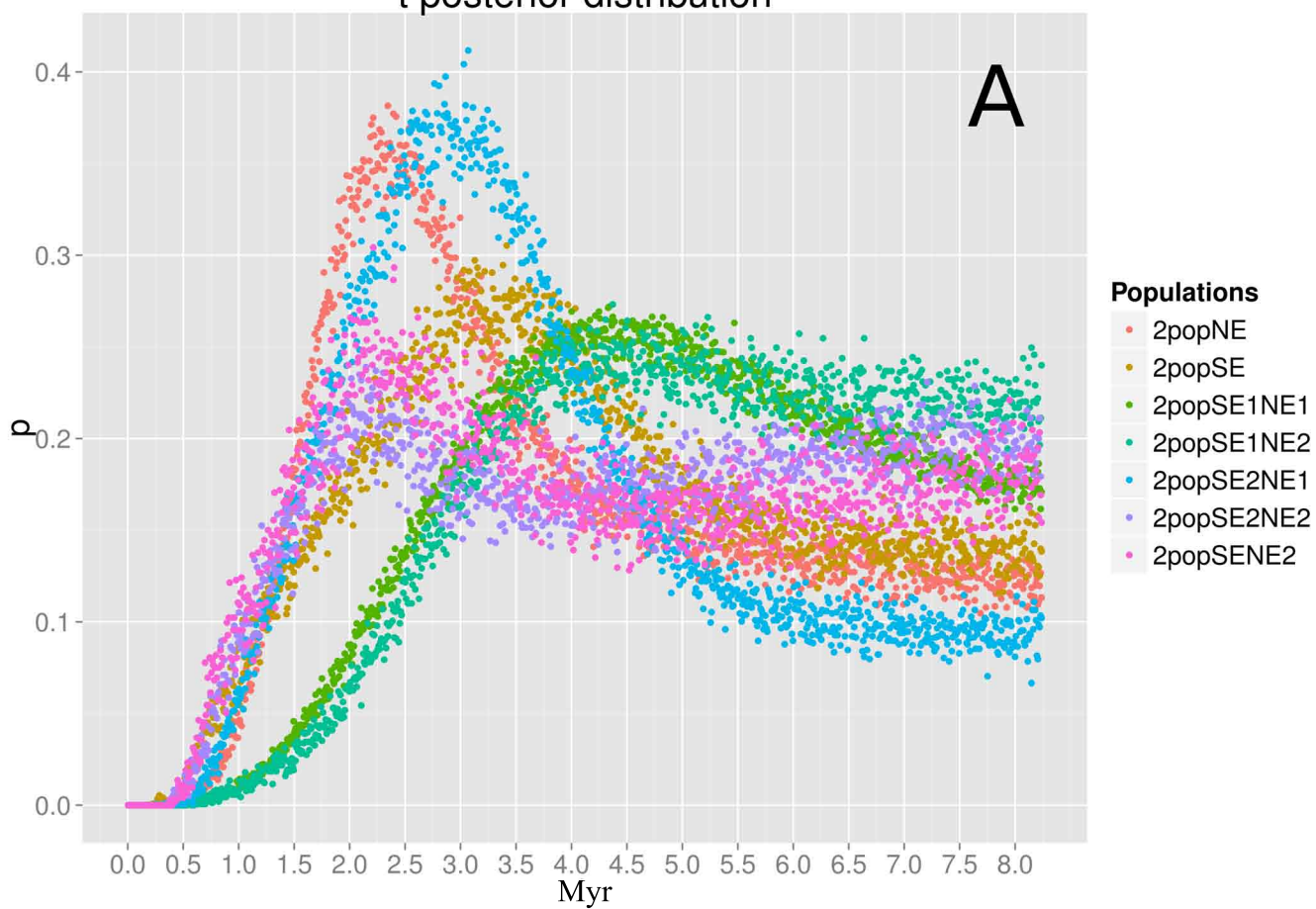

t posterior distribution

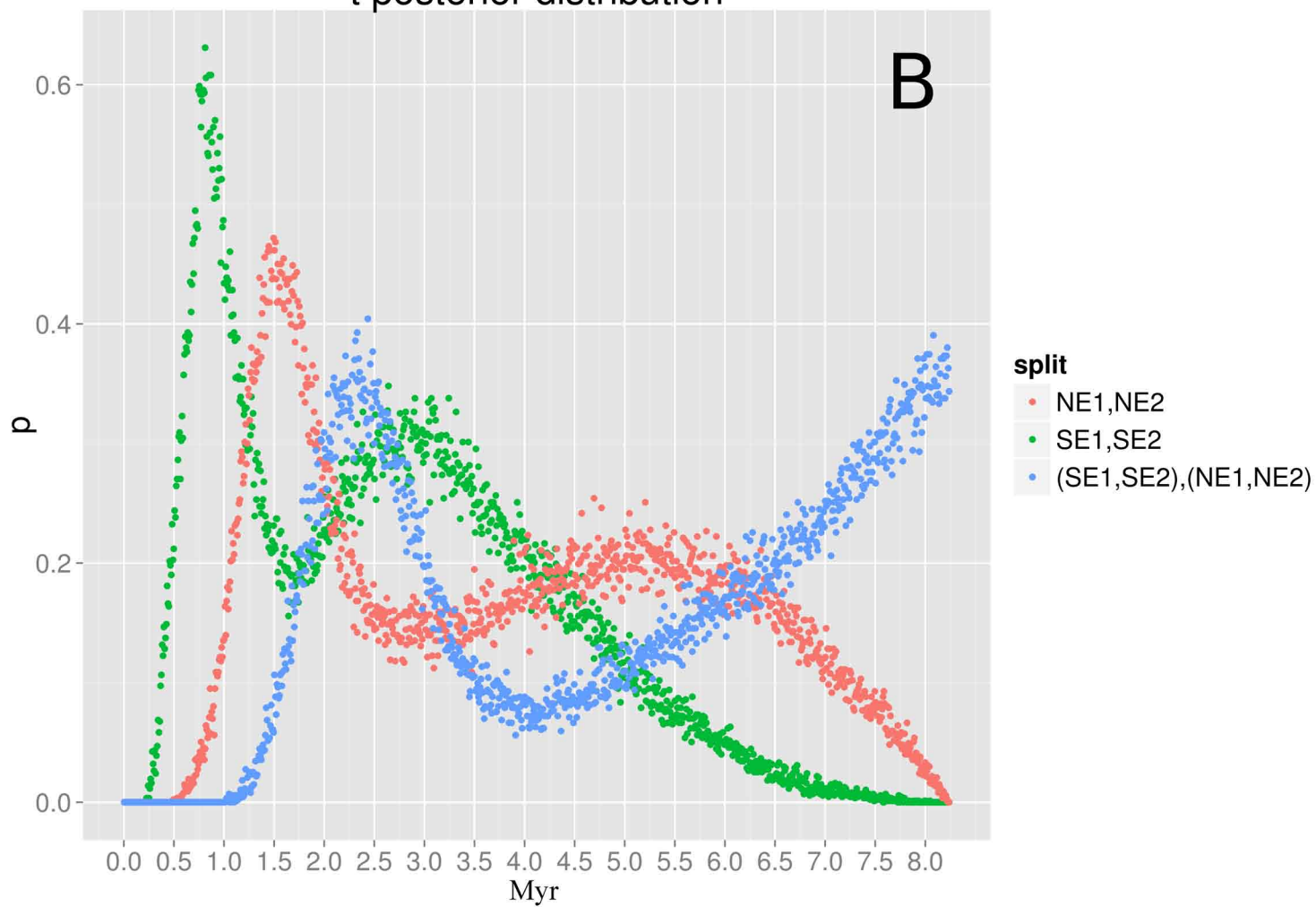

Supplement: S2 Fig — (A) Divergence estimates of the six two-population models. Each curve represents the estimated divergence time between two populations. (B) Divergence time estimates resulting from a four-population model. Each curve represents one of the three divergence parameters of a four-population model. Transformations were performed using the geometric mean of the mutation rates (7.52 x 10^−7^). (PDF) [file pone.0154626.s002.pdf]

Migration rate into NE1

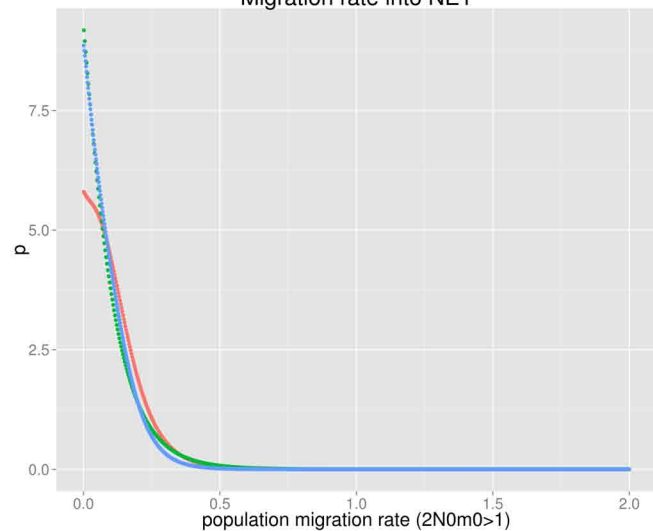

Migration rate into SE1

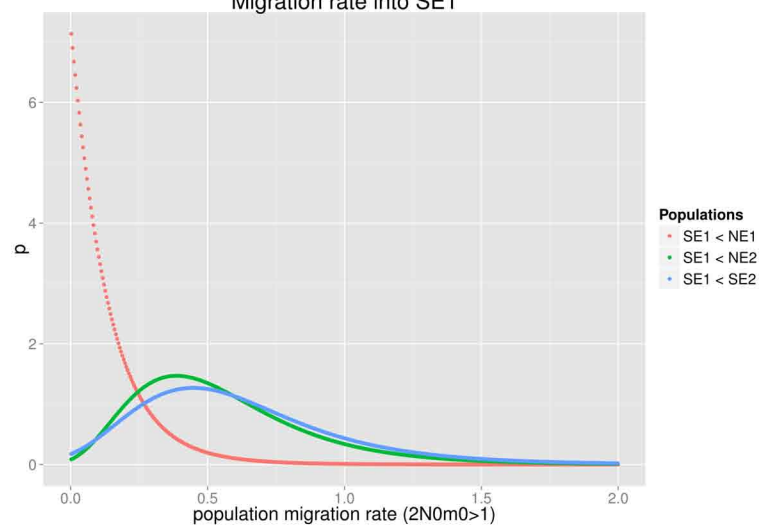

Migration rate into NE2

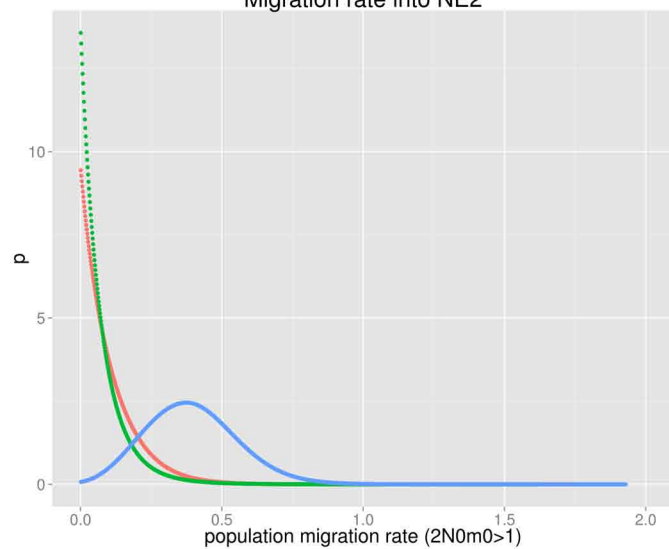

Migration rate into SE2

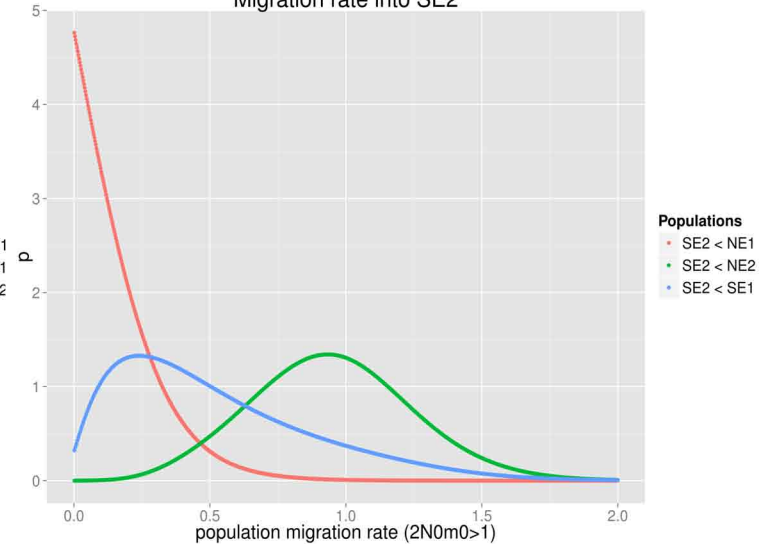

Supplement: S3 Fig — (PDF) [file pone.0154626.s003.pdf]

Migration rate into NE1

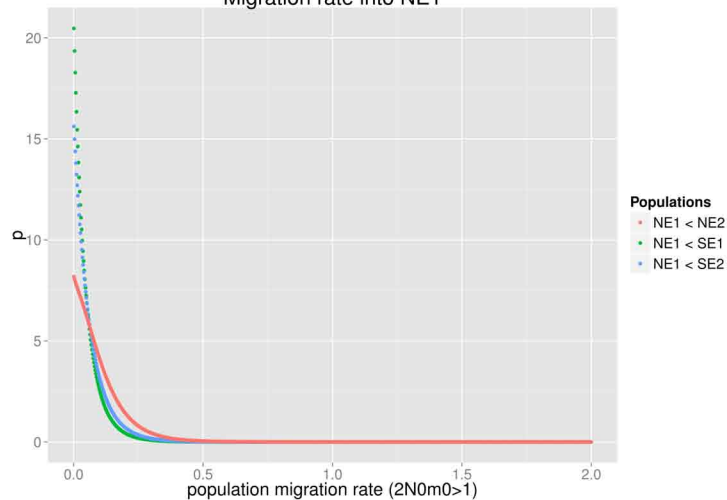

Migration rate into SE1

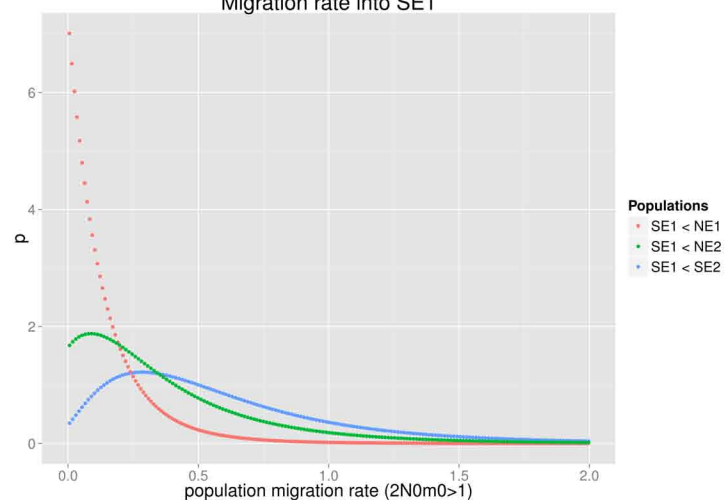

Migration rate into NE2

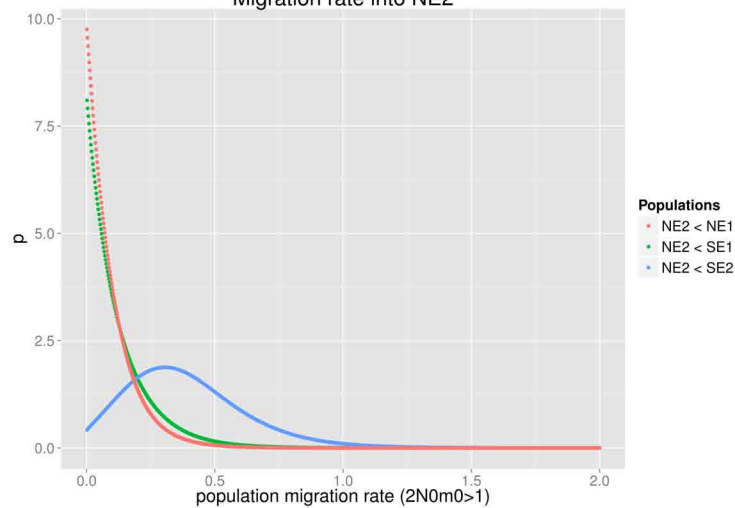

Migration rate into SE2

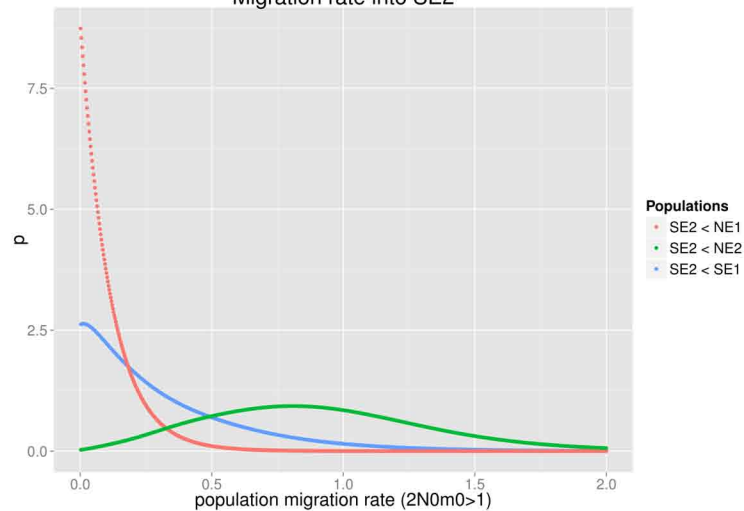

Supplement: S4 Fig — (PDF) [file pone.0154626.s004.pdf]
