# Supplementary material for: Multilocus Phylogeography of the Treefrog Scinax eurydice (Anura, Hylidae) Reveals a Plio-Pleistocene Diversification in the Atlantic Forest
Source: PLoS One. 2016 Jun 1;11(6):e0154626. doi: 10.1371/journal.pone.0154626 (PMC4889069; doi:10.1371/journal.pone.0154626)
Supplement: S5 Fig — Transformations were performed using the geometric mean of the mutation rates (7.52 x 10^−7^). (PDF) [file pone.0154626.s005.pdf]

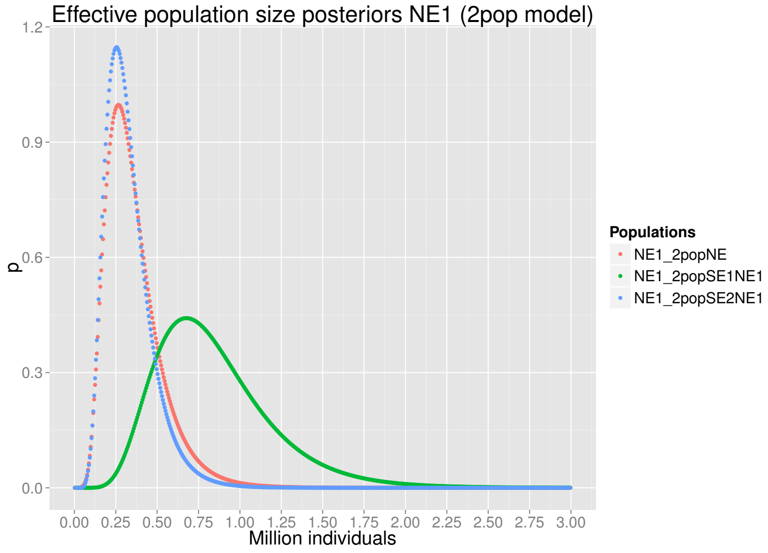
Effective population size posteriors NE1 (2pop model)
p
Million individuals
Populations
NE1_2popNE
NE1_2popSE1NE1
NE1_2popSE2NE1

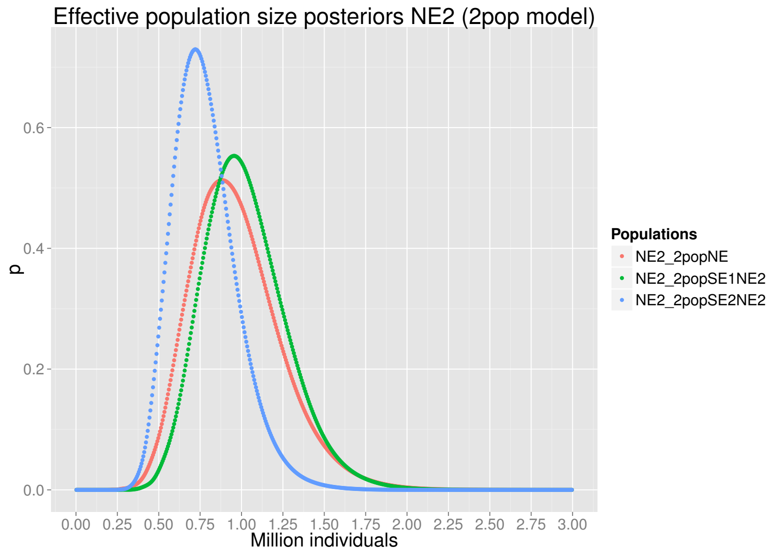
Effective population size posteriors NE2 (2pop model)
p
Million individuals
Populations
NE2_2popNE
NE2_2popSE1NE2
NE2_2popSE2NE2

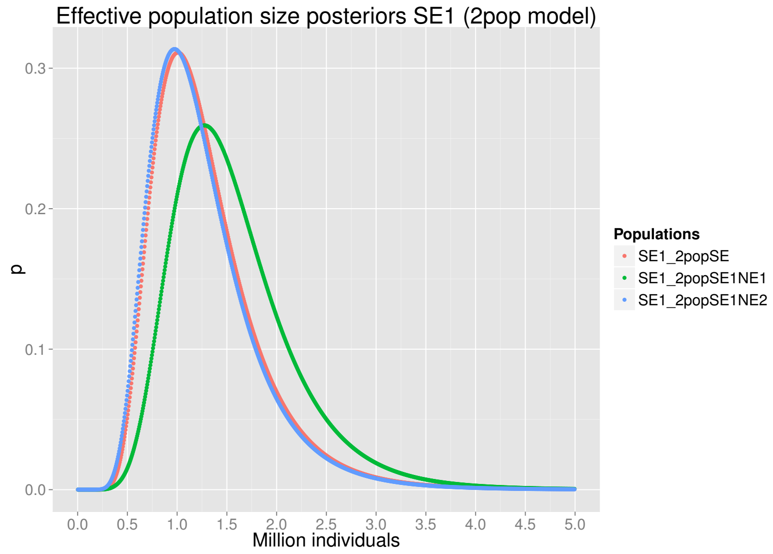
Effective population size posteriors SE1 (2pop model)
p
Million individuals
Populations
SE1_2popSE
SE1_2popSE1NE1
SE1_2popSE1NE2

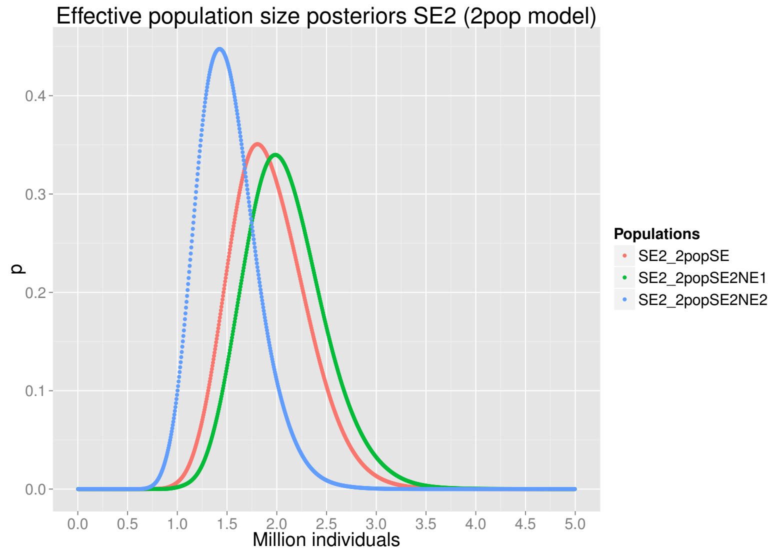
Effective population size posteriors SE2 (2pop model)
p
Million individuals
Populations
SE2_2popSE
SE2_2popSE2NE1
SE2_2popSE2NE2

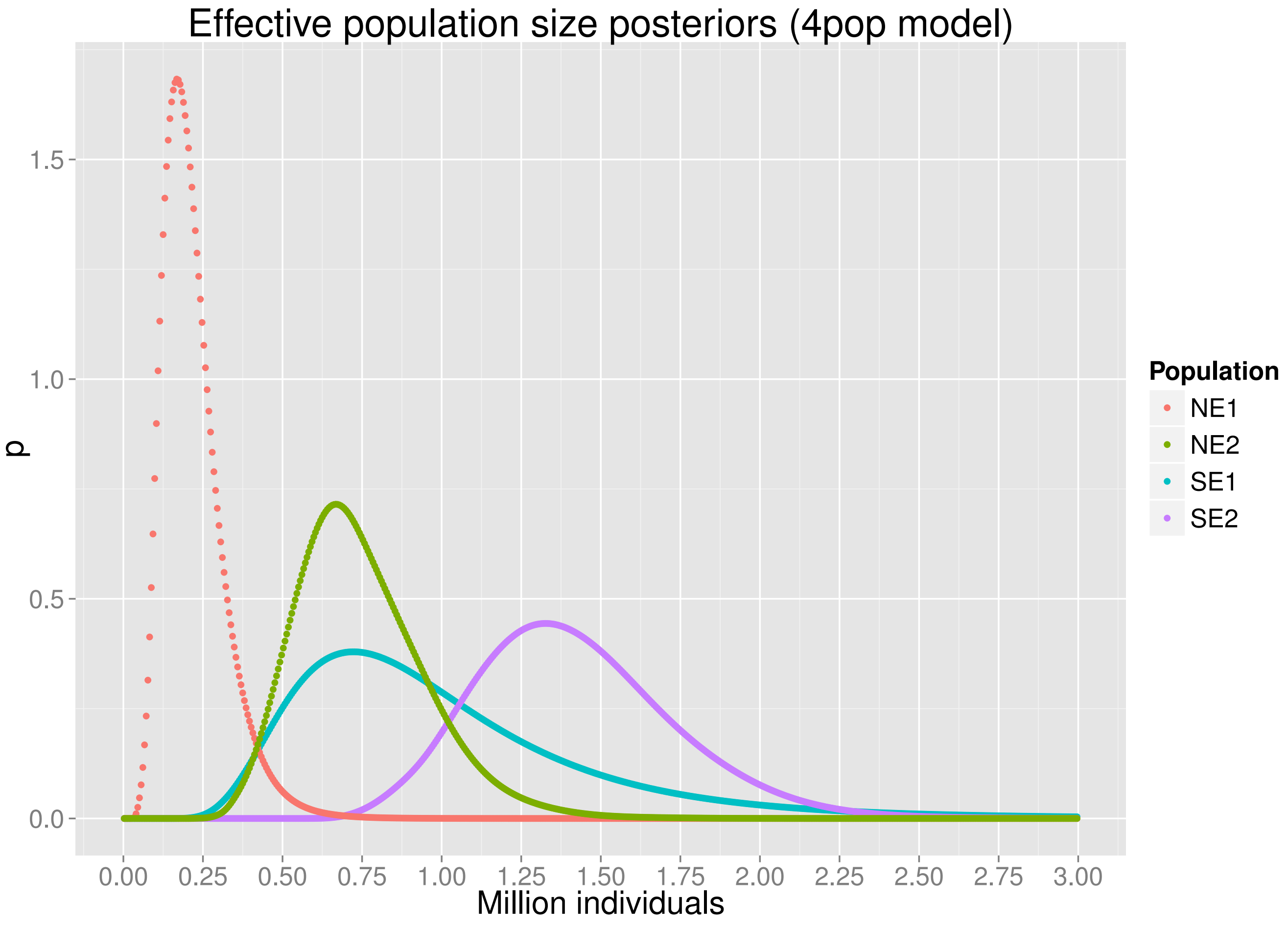
Effective population size posteriors (4pop model)
p
Million individuals
Population
NE1
NE2
SE1
SE2
