# Supplementary material for: Multilocus Phylogeography of the Treefrog Scinax eurydice (Anura, Hylidae) Reveals a Plio-Pleistocene Diversification in the Atlantic Forest
Source: PLoS One. 2016 Jun 1;11(6):e0154626. doi: 10.1371/journal.pone.0154626 (PMC4889069; doi:10.1371/journal.pone.0154626)
Supplement: S6 Fig — Transformations were performed using the geometric mean of the mutation rates (7.52 x 10^−7^). (PDF) [file pone.0154626.s006.pdf]

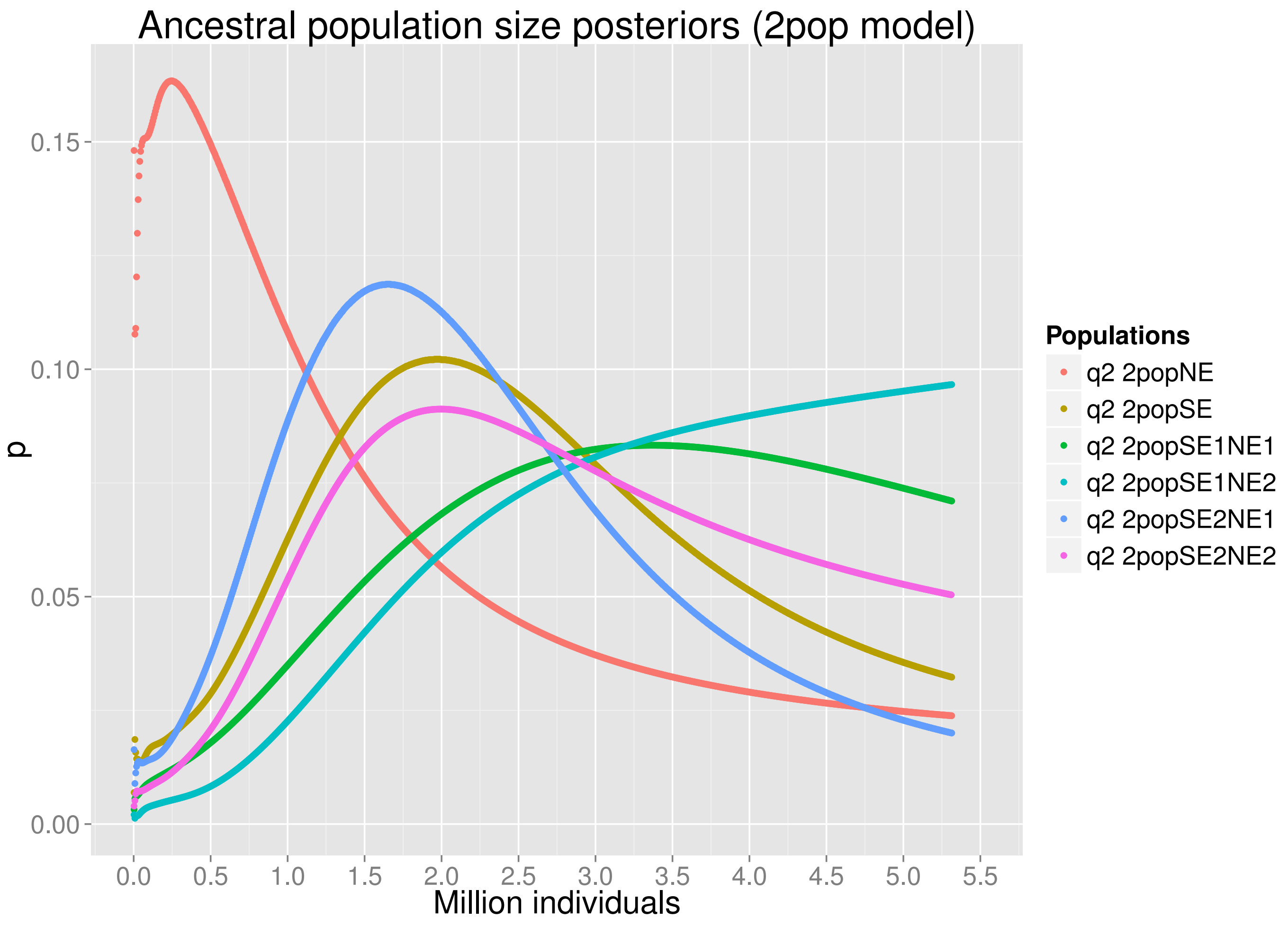

Ancestral population size posteriors (2pop model)
p
0.00
0.05
0.10
0.15
0.0
0.5
1.0
1.5
2.0
2.5
3.0
3.5
4.0
4.5
5.0
5.5
Million individuals
Populations
q2 2popNE
q2 2popSE
q2 2popSE1NE1
q2 2popSE1NE2
q2 2popSE2NE1
q2 2popSE2NE2

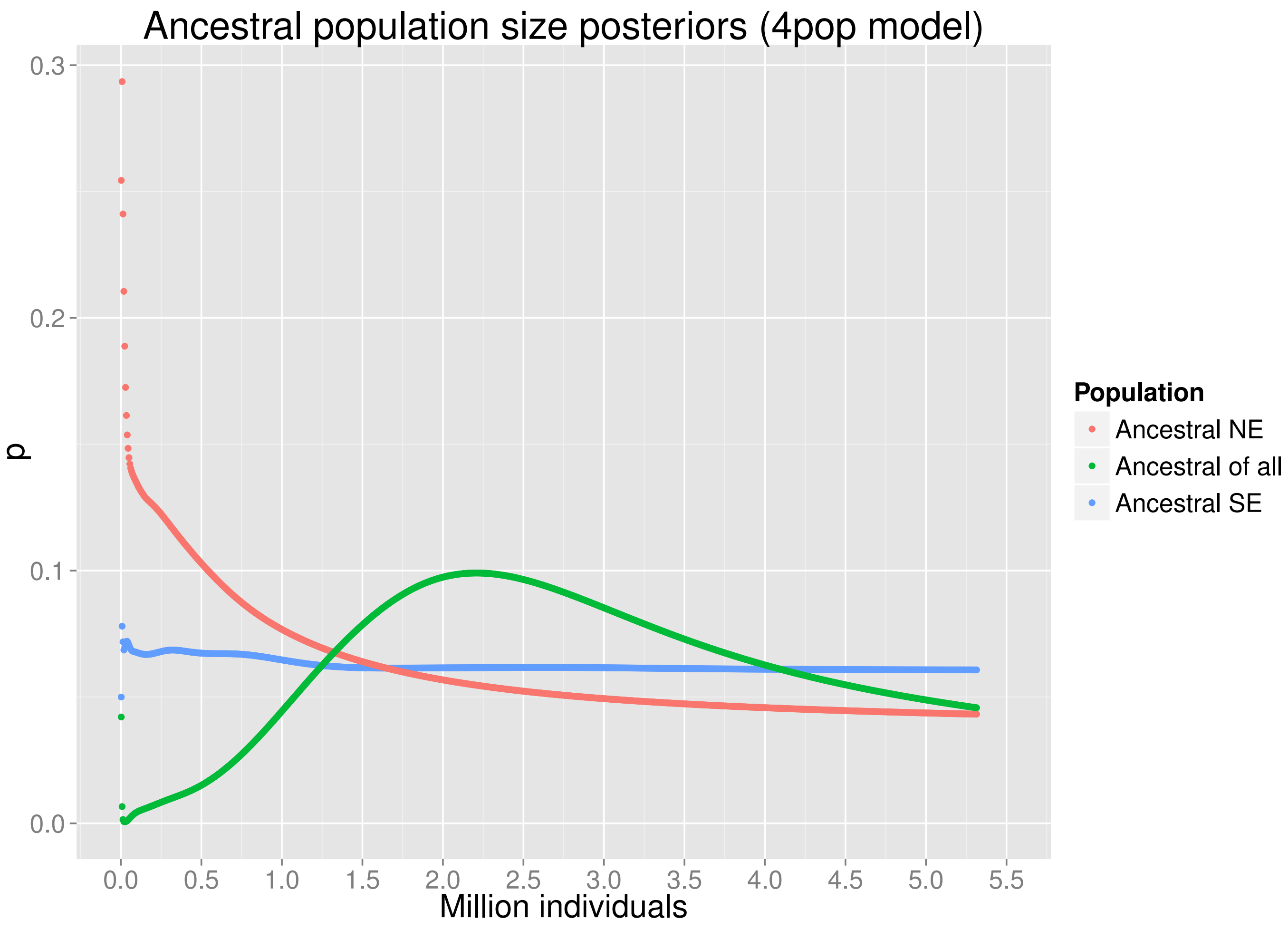

Ancestral population size posteriors (4pop model)
p
0.0
0.1
0.2
0.3
0.0
0.5
1.0
1.5
2.0
2.5
3.0
3.5
4.0
4.5
5.0
5.5
Million individuals
Population
Ancestral NE
Ancestral of all
Ancestral SE
