# Supplementary material for: Multilocus Phylogeography of the Treefrog Scinax eurydice (Anura, Hylidae) Reveals a Plio-Pleistocene Diversification in the Atlantic Forest
Source: PLoS One. 2016 Jun 1;11(6):e0154626. doi: 10.1371/journal.pone.0154626 (PMC4889069; doi:10.1371/journal.pone.0154626)
Supplement: S1 Table — Abbreviations of Brazilian states: PB, Paraíba; PE, Pernambuco; AL, Alagoas; SE, Sergipe; BA, Bahia; ES, Espírito Santo; MG, Minas Gerais; RJ, Rio de Janeiro; SP, São Paulo. Collection acronyms: CHUFPB, Coleção Herpetológica da Universidade Federal da Paraíba; URCA-H, Coleção Herpetológica da Universidade Regional do Cariri; UFBA, Museu de Zoologia da Universidade Federal da Bahia; CFBH; Coleção Célio F.B. Haddad, Departamento de Zoologia, Universidade Estadual Paulista “Júlio de Mesquita Filho”; MNRJ, Museu Nacional, Rio de Janeiro; JC-MTR, LSH-MTR and MTR, Coleção Miguel Trefaut Rodrigues, deposited at the Universidade de São Paulo; PUCMG, Coleção de Herpetologia do Museu de Ciências Naturais da Pontifícia Universidade Católica de Minas; UFMG; Coleção de Herpetologia da Universidade Federal de Minas Gerais; MZUFV, Museu de Zoologia João Moojen, Universidade Federal de Viçosa; IIBP-H; Colección Herpetológica del Instituto de Investigación Biológica del Paraguay, Asunción, Paraguay. (PDF) [file pone.0154626.s007.pdf]

S1 Table. Tissue sample of *Scinax eurydice* used in the account: voucher number, locality, state, coordinates, sequences, haplotypde code and locality code of specimes. Abreviations of brasilian states: PB, Paraíba; PE, Pernambuco; AL, Alagoas; SE, Sergipe; BA, Bahia; ES, Espírito Santo; MG, Minas Gerais; RJ, Rio de Janeiro; SP, São Paulo. Collections acronyms: CHUFPB, Herpetology Collection of Universidade Federal da Paraíba; URCA-H, Herpetology Collection of Universidade Regional do Cariri; UFBA; Museu de Historia Natural da Universidade Federal da Bahia; CFBH; Collection Célio F.B. Haddad, Departamento de Zoologia, Universidade Estadual Paulista "Júlio de Mesquita Filho"; MNRJ, Museu Nacional, Rio de Janeiro; JC-MTR, LSH-MTR and MTR, Miguel T. Rodrigues collection at Universidade de São Paulo; PUCMG, Herpetology Collection of Museu de Ciências Naturais da Pontifícia Universidade Católica de Minas; UFMG; Herpetology Collection of Universidade Federal de Minas Gerais; MZUFV, Museu de Zoologia João Moojen, Universidade Federal da Viçosa; IIBP-H; Herpetology Collection of the Instituto de Investigación Biológica del Paraguay, Asunción, Paraguay.

Voucher number	Locality/State	Coordinates	Sequences	Haplotype	Code
CHUFPB 7956	Guaribas/PB	06º49'S, 35º07'W	ND2; RAG1; 28S	H01	N1
URCA 5081	Pedra D'antas, Lagoa dos Gatos/PE	08º39'S, 35º53'W	ND2; RAG1; B-fibint7; 28S	H02	N2
URCA 5083	Pedra D'antas, Lagoa dos Gatos/PE	08º39'S, 35º53'W	ND2; RAG1; 28S	H03	N2
URCA 4106	Pedra Talhada, Quebrangulo/AL	09º19'S, 36º28'W	ND2; RAG1; 28S	H04	N3
URCA 4181	Pedra Talhada, Quebrangulo/AL	09º19'S, 36º28'W	ND2; RAG1; 28S	H05	N3
URCA 4796	Pedra Talhada, Quebrangulo/AL	09º19'S, 36º28'W	ND2; RAG1; 28S	H06	N3
URCA 5079	Pedra Talhada, Quebrangulo/AL	09º19'S, 36º28'W	ND2; RAG1; 28S	H07	N3
UFBA 210	Jacobina/BA	11º10'S, 40º30'W	RAG1; 28S	H08	N4
UFBA 211	Jacobina/BA	11º10'S, 40º30'W	28S	H09	N4
UFBA 215	Jacobina/BA	11º10'S, 40º30'W	RAG1; 28S	H10	N4
UFBA 213	Jacobina/BA	11º10'S, 40º30'W	RAG1; B-fibint7; 28S	H11	N4
UFBA 214	Jacobina/BA	11º10'S, 40º30'W	RAG1; B-fibint7; 28S	H12	N4
UFBA 217	Jacobina/BA	11º10'S, 40º30'W	RAG1; 28S	H13	N4
UFBA 216	Jacobina/BA	11º10'S, 40º30'W	RAG1; 28S	H14	N4
MNRJ 49743	Indiaroba/SE	11º30'S, 37º30'W	ND2; RAG1; 28S	H15	N5

Voucher number	Locality/State	Coordinates	Sequences	Haplotype	Code
CFBH 13351	Feira de Santana/BA	12º15'S, 38º57'W	ND2; RAG1; B-fibint7; 28S	H16	N6
CFBH 4285	Feira de Santana/BA	12º15'S, 38º57'W	RAG1; B-fibint7; 28S	H17	N6
UFBA 10480	Catu/BA	12º21'S, 38º22'W	ND2; RAG1; B-fibint7; 28S	H18	N7
UFBA 10481	Catu/BA	12º21'S, 38º22'W	ND2; RAG1; B-fibint7; 28S	H19	N7
UFBA 10090	Catu/BA	12º21'S, 38º22'W	ND2; RAG1; B-fibint7; 28S	H20	N7
UFBA 10091	Catu/BA	12º21'S, 38º22'W	ND2; RAG1; B-fibint7; 28S	H21	N7
UFBA 7887	Mata de são João/BA	12º31'S, 38º17'W	ND2; RAG1. B-fibint7; 28S	H22	N8
CFBH 27805	Mata de São João/BA	12º31'S, 38º17'W	ND2; RAG1; 28S	H23	N8
MNRJ 48669	Candeias/BA	12º39'S, 38º33'W	ND2; RAG1, 28S	H24	N9
MTR 19771	Andaraí/BA	12º48'S, 41º19'W	ND2; RAG1; B-fibint7; 28S	H25	N10
MTR 19768	Andaraí/BA	12º48'S, 41º19'W	ND2; RAG1; B-fibint7; 28S	H26	N10
MTR 19769	Andaraí/BA	12º48'S, 41º19'W	ND2; RAG1; B-fibint7; 28S	H27	N10
UFBA 398	Vera Cruz/BA	12º57'S, 38º36'W	RAG1; 28S	H28	N11
JC-MTR 1271	Mucugê/BA	13º00'S, 41º22'W	ND2; RAG1; 28S	H29	N12
UFBA 7297	Amargosa/BA	13º01'S, 39º36'W	RAG1; B-fibint7; 28S	H30	N13
UFBA 3260	Maracás/BA	13º26'S, 40º25'W	RAG1; B-fibint7; 28S	H31	N14
UFBA 2361	Maracás/BA	13º26'S, 40º25'W	RAG1; B-fibint7; 28S	H32	N14
UFBA 2366	Maracás/BA	13º26'S, 40º25'W	RAG1; B-fibint7; 28S	H33	N14
UFBA 2362	Maracás/BA	13º26'S, 40º25'W	RAG1; B-fibint7; 28S	H34	N14
UFBA 2365	Maracás/BA	13º26'S, 40º25'W	RAG1; B-fibint7; 28S	H35	N14
UFBA 2364	Maracás/BA	13º26'S, 40º25'W	RAG1; 28S	H36	N14

Voucher number	Locality/State	Coordinates	Sequences	Haplotype	Code
UFBA 2367	Maracás/BA	13º26'S, 40º25'W	RAG1; B-fibint7; 28S	H37	N14
UFBA 2372	Maracás/BA	13º26'S, 40º25'W	RAG1; 28S	H38	N14
UFBA 2371	Maracás/BA	13º26'S, 40º25'W	RAG1; B-fibint7; 28S	H39	N14
MNRJ 46507	Boa Nova/BA	14º21'S, 40º12'W	RAG1; B-fibint7; 28S	H40	N15
CFBH 23370	Uruçuca/BA	14º35'S, 39º16'W	ND2; B-fibint7; 28S	H41	N16
CFBH 23372	Uruçuca/BA	14º35'S, 39º16'W	ND2; RAG1; B-fibint7	H42	N16
CFBH 23371	Uruçuca/BA	14º35'S, 39º16'W	B-fibint7; 28S	H43	N16
CFBH 23369	Uruçuca/BA	14º35'S, 39º16'W	ND2; RAG1; B-fibint7; 28S	H44	N16
CFBH 2884	Itabuna/BA	14º46'S, 39º16'W	ND2; RAG1, B-fibint7; 28s	H48	N18
UFBA 7480	Ilhéus/BA	14º46'S, 30º20'W	ND2; RAG1; B-fibint7; 28S	H45	N17
UFBA 7481	Ilhéus/BA	14º46'S, 30º20'W	RAG1; B-fibint7; 28S	H46	N17
MNRJ 51709	Ilhéus/BA	14º46'S, 30º20'W	ND2; RAG1; 28S	H47	N17
MTR 17302	Jequitinhonha/MG	16º25'S, 41º00'W	ND2; RAG1; B-fibint7; 28S	H49	S1
MTR 17303	Jequitinhonha/MG	16º25'S, 41º00'W	ND2; B-fibint7; 28S	H50	S1
MTR 17424	Jequitinhonha/MG	16º25'S, 41º00'W	ND2; RAG1; B-fibint7; 28S	H51	S1
CFBH 10237	Grão Mogol/MG	16º33'S, 42º52'W	ND2; RAG1; B-fibint7; 28S	H52	S2
PUCMG 170	Grão Mogol/MG	16º33'S, 42º52'W	ND2; RAG1; B-fibint7; 28S	H53	S2
CFBH 7690	Prado/BA	17º20'S, 39º13'W	ND2; RAG1; B-fibint7; 28S	H54	N19
UFMG 9583	São João Evangelista/MG	18º32'S, 42º45'W	ND2; RAG1; 28S	H55	S3
MTR 19619	PARNA Serra do Cipó/MG	19º12'S, 43º26'W	ND2; RAG1; B-fibint7; 28S	H56	S4
MTR 19620	PARNA Serra do Cipó/MG	19º12'S, 43º26'W	ND2; RAG1; B-FIBINT7; 28S	H57	S4

Voucher number	Locality/State	Coordinates	Sequences	Haplotype	Code
UFMG 5415	Morro do Pilar/MG	19º12'S, 43º22'W	ND2; RAG1; 28S	H58	S5
MTR 12048	Linhares/ES	19º23'S, 40º03'W	ND2; RAG1; 28S	H59	S6
MTR 12096	Linhares/ES	19º23'S, 40º03'W	ND2; RAG1; 28S	H60	S6
MTR 12172	Linhares/ES	19º23'S, 40º03'W	ND2; RAG1; B-fibint7; 28S	H61	S6
MTR 17551	Marliéria/MG	19º42'S, 42º43'W	ND2; RAG1; 28S	H62	S7
MTR 17552	Marliéria/MG	19º42'S, 42º43'W	ND2; RAG1; B-fibint7; 28S	H63	S7
CFBH 14498	Aracruz/ES	19º48'S, 40º16'W	ND2; RAG1; B-fibint7; 28S	H64	S8
UFMG 1176	São Gonçalo do Rio Abaixo/MG	19º49'S, 43º21'W	ND2; RAG1; B-fibint7; 28S	H65	S9
CFBH 373	Santa Teresa/ES	19º55'S, 40º35'W	ND2; RAG1; B-fibint7; 28S	H66	S10
CFBH 374	Santa Teresa/ES	19º55'S, 40º35'W	ND2; RAG1; B-fibint7; 28S	H67	S10
UFMG 5197	Catas Atlas/MG	20º04'S, 43º24'W	ND2; RAG1; B-fibint7; 28S	H68	S11
MNRJ 77773	Catas Atlas/MG	20º04'S, 43º24'W	ND2; RAG1; 28S	H69	S11
MTR 12601	Parque Nacional do Caparaó/ES	20º14'S, 41º28'W	ND2; RAG1; B-fibint7; 28S	H70	S12
CFBH 13313	Viçosa/MG	20º45'S, 42º52'W	ND2; RAG1; 28S	H71	S13
CFBH 13315	Viçosa/MG	20º45'S, 42º52'W	ND2; 28S	H72	S13
MZUFV 12841	Viçosa/MG	20º45'S, 42º52'W	ND2; RAG1; B-fibint7; 28S	H73	S13
CFBH 13316	Viçosa/MG	20º45'S, 42º52'W	ND2; RAG1; 28S	H74	S13
CFBH 13324	Viçosa/MG	20º45'S, 42º52'W	RAG1; B-fibint7; 28S	H75	S13
CFBH 13322	Viçosa/MG	20º45'S, 42º52'W	RAG1; 28S	H76	S13
MZUFV 12821	Viçosa/MG	20º45'S, 42º52'W	ND2; RAG1; 28S	H77	S13
MZUFV 12843	Viçosa/MG	20º45'S, 42º52'W	ND2; RAG1; 28S	H78	S13

Voucher number	Locality/State	Coordinates	Sequences	Haplotype	Code
MZUFV 12841	Viçosa/MG	20º45'S, 42º52'W	ND2; RAG1; B-fibint7; 28S	H79	S13
MZUFV 12842	Viçosa/MG	20º45'S, 42º52'W	ND2; RAG1; B-fibint7; 28S	H80	S13
MZUFV 12823	Viçosa/MG	20º45'S, 42º52'W	ND2; RAG1; B-fibint7; 28S	H81	S13
MZUFV 12822	Viçosa/MG	20º45'S, 42º52'W	ND2; RAG1; B-fibint7; 28S	H82	S13
LSH-MTR 64	Itapemirim/ES	21º00'S, 40º49'W	ND2; RAG1; B-fibint7; 28S	H83	S14
UFMG 6331	Lima Duarte/MG	21º50'S, 43º47'W	ND2; RAG1; B-fibint7; 28S	H84	S15
MNRJ 63723	Trajano de Moraes/RJ	22º03'S, 43º03'W	ND2; RAG1; B-fibint7; 28S	H85	S16
CFBH 13937	Petrópolis/RJ	22º30'S, 43º10'W	ND2; RAG1; B-fibint7; 28S	H86	S17
CFBH 14210	São José do Barreiro/SP	22º38'S, 44º34'W	RAG1; B-fibint7; 28S	H87	S18
MNRJ 53752	Magé/RJ	22º39'S, 43º02'W	ND2; RAG1; 28S	H88	S19
MNRJ 53751	Magé/RJ	22º39'S, 43º02'W	ND2; RAG1; 28S	H89	S19
CFBH 16736	Jundiaí/SP	23º10'S, 46º52'W	ND2; RAG1; 28S	H90	S20
CFBH 7706	Jundiaí/SP	23º10'S, 46º52'W	ND2; RAG1; 28S	H91	S20
CFBH 5781	Parati/RJ	23º12'S, 44º42'W	ND2; RAG1; B-fibint7; 28S	H92	S21
CFBH 17624	Ubatuba/SP	23º25'S, 45º04'W	ND2; RAG1; B-fibint7; 28S	H93	S22
CFBH 7	Ubatuba/SP	23º25'S, 45º04'W	ND2; RAG1; 28S	H94	S22
UFBA 11703	*S. x-signatus*	-	ND2; RAG1; 28S	Out group	-
CFBHT 10951	*S. nebulosus*	-	ND2	Out group	-
IIBP-H 1396	*S. berthae*	-	ND2	Out group	-
UFBA 7893	*S. auratus*	-	ND2	Out group	-
