# Supplementary material for: Multilocus Phylogeography of the Treefrog Scinax eurydice (Anura, Hylidae) Reveals a Plio-Pleistocene Diversification in the Atlantic Forest
Source: PLoS One. 2016 Jun 1;11(6):e0154626. doi: 10.1371/journal.pone.0154626 (PMC4889069; doi:10.1371/journal.pone.0154626)
Supplement: S2 Table — (PDF) [file pone.0154626.s008.pdf]

S2 Table. Gene fragment, primer, sequence of primer and reference used in this study.

Fragment	Primer	Sequece primer (M13+primer)	References
28S	M13+28SV	5’- TGTAAAACGACGGCCAGTAAGGTAGCCAAATGCCTCGTCATC-3’	Hillis and Dixon (1991)
	M13+28SSJ	5’- GAGCGGATAACAATTTCACACAGGAGTAGGGTAAAACTAACCT-3’	Hillis and Dixon (1991)
β-fibint7	M13+FIB_B17L	5' - GTAAAACGACGGCCAGTTCCCCAGTAGTATCTGCCATTAGGGTT - 3'	Prychitko and Moore (1997)
	M13+FIB_B17U	5' - GCGGATAACAATTTCACACAGGGGAGAAAACAGGACAATGACAATTCAC - 3'	Prychitko and Moore (1997)
RAG1	M13+R1-GFF	5' - GTAAAACGACGGCCAGTGAGAAGTCTACAAAAAVGGCAAAG - 3'	T. Grant & J. Faivovich
	M13+R1-GFR	5' - GCGGATAACAATTTCACACAGGGAAGCGCCTGAACAGTTTATTAC - 3'	T. Grant & J. Faivovich
ND2	M13+L4437	5' - GTAAAACGACGGCCAGTAAGCTTTCGGGCCCATACC - 3'	Macey *et al.* (1997)
	M13+Int_Halbo2	5' – GCGGATAACAATTTCACACAGGGTCTAATTTATCCTAAGTTTC - 3'	Prado *et al.* (2012)
