# Supplementary material for: Multilocus Phylogeography of the Treefrog Scinax eurydice (Anura, Hylidae) Reveals a Plio-Pleistocene Diversification in the Atlantic Forest
Source: PLoS One. 2016 Jun 1;11(6):e0154626. doi: 10.1371/journal.pone.0154626 (PMC4889069; doi:10.1371/journal.pone.0154626)
Supplement: S3 Table — (PDF) [file pone.0154626.s009.pdf]

S3 Table. K run numbers, replicates, mean of log-likelihood and standard deviation of log-likelihood.

K	Replicates	Mean est, LnP (data)	Stdev est, LnP (data)
2	10	-728.16	0.069921
3	10	-692.49	0.196921
4	10	-692.38	0.042164
5	10	-705.80	0.105409
6	10	-718.98	0.181353
7	10	-732.43	0.211082
8	10	-745.17	0.266875
9	10	-757.51	0.166333
10	10	-769.55	0.313581
