# Supplementary material for: Multilocus Phylogeography of the Treefrog Scinax eurydice (Anura, Hylidae) Reveals a Plio-Pleistocene Diversification in the Atlantic Forest
Source: PLoS One. 2016 Jun 1;11(6):e0154626. doi: 10.1371/journal.pone.0154626 (PMC4889069; doi:10.1371/journal.pone.0154626)
Supplement: S4 Table — (PDF) [file pone.0154626.s010.pdf]

S4 Table. Parameters of posterior distributions of population migration rate estimation for two and four population models. * P < 0.05; ** P < 0.01; *** P < 0.001.

Populations in the model	**SE1<SE2**		**SE1<NE1**		**SE1<NE2**		**SE2<SE1**		**SE2<NE1**		**SE2<NE2**		**NE1<SE1**		**NE1<SE2**		**NE1<NE2**		**NE2<SE1**		**NE2<SE2**		**NE2<NE1**	
	two	*four*	*two*	*Four*	*Two*	*four*	*two*	*four*	*two*	*four*	*two*	*four*	*two*	*four*	*two*	*four*	*two*	*four*	*two*	*four*	*two*	*four*	*two*	*four*
HiPt	0.449	0.282	0.002	0.005	0.386	0.090	0.240	0.014	0.001	0.001	0.932	0.808	0.002	0.001	0.001	0.001	0.001	0.001	0.001	0.001	0.374	0.307	0.001	0.001
Mean	0.626	0.623	0.141	0.153	0.563	0.406	0.525	0.334	0.174	0.113	0.948	0.913	0.103	0.052	0.091	0.066	0.116	0.099	0.079	0.126	0.393	0.391	0.103	0.099
95%Lo	0.089	0.054	0.002	0.005	0.103	0.018	0.049	0.008	0.006	0.004	0.390	0.196	0.002	0.001	0.003	0.001	0.004	0.004	0.002	0.004	0.103	0.044	0.003	0.003
95%Hi	1.581	1.973	0.539	0.622	1.408	1.469	1.398	1.180	0.578	0.419	1.568	1.897	0.379	0.208	0.291	0.251	0.353	0.339	0.311	0.469	0.735	0.933	0.364	0.364
Significance	*	*			***		*				***	**									**	*		
