# Supplementary material for: Multilocus Phylogeography of the Treefrog Scinax eurydice (Anura, Hylidae) Reveals a Plio-Pleistocene Diversification in the Atlantic Forest
Source: PLoS One. 2016 Jun 1;11(6):e0154626. doi: 10.1371/journal.pone.0154626 (PMC4889069; doi:10.1371/journal.pone.0154626)
Supplement: S1 Appendix — (PDF) [file pone.0154626.s011.pdf]

S1 Appendix. Laboratory procedures.

Genomic DNA was extracted from liver or muscle samples using PureLink extraction kit (Invitrogen). We amplified and sequenced fragments of one mitochondrial gene and three nuclear genes. The mitochondrial gene corresponded to the partial NADH dehydrogenase fragment of subunit 2 (ND2, 969 bp). The nuclear fragments corresponded to the recombination activation gene (RAG1, 429 bp), the β-fibrinogen intron 7 (β-fibint7, 321 bp) and the 28S ribosomal RNA (28S, 796 bp). We used GoTaq Green Master Mix 2X (Promega) and primers specific for each gene (see Table S2) plus M13 tail for fragment amplification. PCR conditions for ND2 were as follow: (i) initial phase of 2 min at 94 °C; (ii) 25 cycles, each cycle consisting of 30 seconds at 94 °C, 30 seconds at 56 °C, 2 min at 72 °C; and (iii) final extension of 7 minutes at 72 °C. PCR conditions for nuclear markers were as follow: (i) initial phase of 2 min at 94 °C; (ii) 34-45 cycles, each cycle consisting of 30 seconds at 94°C, 30 seconds at 52-54 ºC and 2 minutes at 72 °C; and (iii) final extension of 7 minutes at 72 °C. The products obtained by PCR were visualized on 1-2% agarose gel. Purification and sequencing were performed by High Throughput Genomics Center, Seattle, USA (HTSeq).

Electropherograms were inspected and assembled in contigs using CodonCode Aligner v. 3.5 (CodonCode Inc.). Heterozygous sites in nuclear introns were coded according to IUPAC code when double peaks were present in both strands of the same individual's electropherograms.
