# Supplementary material for: Multilocus Phylogeography of the Treefrog Scinax eurydice (Anura, Hylidae) Reveals a Plio-Pleistocene Diversification in the Atlantic Forest
Source: PLoS One. 2016 Jun 1;11(6):e0154626. doi: 10.1371/journal.pone.0154626 (PMC4889069; doi:10.1371/journal.pone.0154626)
Supplement: S2 Appendix — (PDF) [file pone.0154626.s012.pdf]

S2 Appendix. IMa2 Divergence Times Estimation Results.

IMa2 split estimates for the two-population models show a unimodal distribution with a plateau towards the end of the distribution (S2 Fig). In all two-population model cases divergence estimates fail to reach zero probabilities at the upper prior bound. Lower 95 high posterior densities of the divergence between NE1 and NE2 and SE1 and SE2 are around 1.0 Myr with a peak around 2.2 and 3.3 Myr respectively (S2 Fig). These old estimates may result from migration of one of the two populations to a third, more divergent population, which is the case here (see 'migration' results section). In the four-population analysis, divergence estimates between northeastern and southeastern populations shows a bimodal distribution with a more recent higher density peak followed by a second peak with lower density in both cases.

Therefore, these results should be considered with caution and can be interpreted as two possible divergence scenarios, one with a more recent divergence and lower migration and another with an older divergence and higher migration [51]. First peak estimates largely agree with BEAST estimates with a divergence of around 0.8 Myr for SE1 and SE2 and 1.5 Myr for NE1 and NE2 (S2 Fig). Second peaks suggest a divergence around 3 Myr between SE1 and SE2; and around 5 Myr between NE1 and NE2 (S2 Fig). Confidence intervals are not reliable due to the double peak densities. The posterior distribution of the divergence between the northeastern ancestral population and the ancestral population of the southeaster populations presents a peak with a raising tail towards the upper limit of the prior. The peak estimate is also in agreement with the species tree analysis with a divergence date around 2.3 Myr.

References

51. Strasburg JL, Rieseberg LH. How robust are "isolation with migration" analyses to violations of the im model? A simulation study. Molecular Biology and Evolution. 2010;27: 297-310.
